# An Augmented Reality and AI-Based System for Contextual Appliance Guidance: Implications for Cognitive Accessibility and Assistive Interaction

**DOI:** 10.3390/brainsci16080783

**Published:** 2026-07-24

**Authors:** Kimia Hafezi, Atra Hossein Tafreshi, Christian Napoli, Cristian Randieri, Samuele Russo

**Affiliations:** 1 Department of Computer, Control, and Management Engineering “Antonio Ruberti”, Sapienza University of Rome, 00185 Rome, Italy; hafezi.1908578@studenti.uniroma1.it (K.H.); hosseintafreshi.1908579@studenti.uniroma1.it (A.H.T.); cnapoli@diag.uniroma1.it (C.N.); 2 Department of Artificial Intelligence, Czestochowa University of Technology, ul. Dąbrowskiego 69, 42-201 Czestochowa, Poland; 3 Department of Theoretical and Applied Sciences, eCampus University, Via Isimbardi 10, 22060 Novedrate, Italy; samuele.russo@uniroma1.it

**Keywords:** augmented reality, visual sensing, YOLOv8, assistive technology, appliance usability, language models, human-computer interaction, accessibility, cognitive impairment, user manuals

## Abstract

Background: Modern household appliances often present complex interfaces that can be difficult to use, especially for older adults, people with visual impairments, and users with mild cognitive difficulties. In such cases, interacting with appliances may require sustained attention, visuospatial search, working memory, and sequential action planning, while traditional user manuals often provide limited contextual support. Methods: To address this issue, this study presents a proof-of-concept augmented reality (AR) and artificial intelligence (AI)-based system for contextual appliance guidance. The proposed architecture integrates visual sensing, deep learning, and large language models to detect appliance controls, interpret user queries, retrieve relevant information from user manuals, and provide step-by-step guidance directly on the real interface. A YOLOv8 model trained on a custom dataset was used for button detection, YOLO-Seg was employed to enhance visual highlighting through segmentation, and BoT-SORT was used to maintain detection consistency across frames. A Unity-based mobile application displayed real-time AR overlays with customizable visual settings for accessibility needs, such as low vision and color blindness that may be relevant for future accessibility-oriented applications. In addition to its technical pipeline, the system is conceptually relevant as a potential form of external cognitive support because it transforms static manual instructions into situated, sequential, and visually grounded guidance.Results: Experimental results showed promising technical performance for button detection and segmentation, while a preliminary user evaluation in a non-clinical sample suggested good usability, clarity, and acceptability of the interface. Conclusions: These findings support the technical feasibility and preliminary usability of the approach, while cognitive workload, confidence, functional autonomy, and clinical benefit were not directly measured. Targeted validation in older adults, people with visual impairments, and clinical populations is therefore still needed. Future developments will include multimodal feedback, read-aloud guidance, and more specific evaluation of workload, confidence, and functional autonomy.

## 1. Introduction

The increasing diversity of home and office appliances and the complexity of their usage have introduced new challenges for users. Recent studies have highlighted this issue. A survey conducted by OnePoll on behalf of Beko in October 2022, which included a sample of 2000 adults, shows that nearly half of the participants rarely change the settings of their appliances due to fear of causing damage or lack of knowledge of the function. Specifically, 44% of users reported that they prefer to stick to the settings they are familiar with because they know the result, and 20% don’t have a clue what the other settings do, so they just ignore them entirely. According to the same survey, 55% of participants felt overwhelmed by the number of logos and labels on their home appliances; therefore, they ended up using the wrong setting or energy in inefficient settings [1]. In addition to general usability concerns, these challenges are more substantial for older people or people with visual impairments, and mild cognitive impairments that affect a significant portion of the aging population, with an estimated 12–18% of individuals aged 60 or older living with this condition [2]. As new appliances contain a lot of complex settings, they require recalling button functions, following multi-step procedures, and adjusting settings accurately. For older adults with MCI (Mild Cognitive Impairment), memory challenges often lead to forgetting the sequence of operations and the correct appliance settings or safety steps. Language difficulties are another barrier for users with MCI, because they need to read the labels and interpret user manuals to use the appliances. Furthermore, the likelihood of selecting incorrect settings increases for them due to impaired decision-making. Moreover, spatial and visual issues make it difficult for these patients to locate and distinguish the buttons and interface elements [3].

While there are user manuals provided by manufacturers, which contain information about the operation and settings of appliances, searching for them and following their instructions is not easy. Also, the accessibility of user manuals in traditional forms is an issue, and they only have some predefined instructions, without the flexibility to guide users based on their specific needs. This limitation makes the user experience inefficient and limits their ability to benefit from the new technological advancements in appliances.

To address these limitations, we propose a proof-of-concept AR-based guidance prototype that integrates deep learning models for appliance-control detection, a language model for instruction generation, and an augmented reality interface to support contextual appliance guidance. This integrated design is intended to explore the technical feasibility of combining these components across different appliances and interaction scenarios. First, appliance buttons are detected and classified by a fine-tuned object detection model that uses a prepared dataset. The system uses visual sensing through the mobile device’s camera to capture real-time images of the appliance interface for analysis and guidance. Second, these detected buttons are analyzed by the language model to determine their attributes, including the button name, relative position on the appliance, appearance, and functionality. The system provides step-by-step guidance and instruction by using the language model. Third, developing an augmented reality application facilitates the visualization of the highlighted buttons to push and provides instructional information in real time. Fourth, integrating whole pieces together to have seamless functionality. While the standard user manuals provide only general appliance guidance, in this work, the language model can adjust the instructions for specific needs. For instance, if a user has a question about how to bake a chocolate cake in a specific oven model, the system, in addition to providing the settings and temperatures, gives all the information needed from a recipe, all the cooking steps, and the safety guidelines, and assists the user visually and textually during the process of baking a cake.In this scenario, recipe-related information and appliance-specific guidance are treated as different sources of information. Recipe steps can support the cooking task, while appliance settings, button usage, and safety-related instructions are grounded in the retrieved user manual and controlled through backend prompts. This feature, especially, has the benefit for users with a cognitive impairment since it eliminates the need to memorize the steps and allows the user to follow the instructions step-by-step in real time, and furthermore, there is an option to customize the color and contrast of the overlays based on the user’s needs.

By integrating augmented reality (AR) in the mobile application, there may be some improvements in the usability and effectiveness of the interaction between the user and the application, especially when the users are older adults, people with cognitive or visual difficulties, or users with memory-related challenges. AR-based guidance may help users interact with the appliances by three main factors. The first one is that when using AR guidance, there is less need to read the complex user manuals, and AR instead can highlight the necessary buttons directly on the appliance, so the interaction can be easier to follow. Second, this system gives real-time guidance to the user and helps the user follow the steps. Finally, with the help of interactive feedback, users may feel more confident during the task, although this effect was not directly measured in the present study. For people with low vision or color blindness, the benefits of this system can be highlighting the buttons, and the interface contrast can be adjusted in a way that is suitable for users with low vision. In conclusion, the AR system with voice and customized high-contrast overlays may support future accessibility-oriented applications.

Since users with early-stage Alzheimer’s disease or other memory-related difficulties may experience problems with recall and sequential task execution, following written instructions can be particularly challenging. AR-based applications may help by providing step-by-step guidance in visual and audio formats, thereby reducing the need to retain the full sequence of actions in memory at once. However, these possible benefits were not evaluated with clinical users in this study and should be considered as hypotheses for future validation.

From a psychological perspective, the relevance of such a system extends beyond interface convenience. The use of modern appliances often depends on a combination of cognitive functions, including selective attention, visuospatial search, working memory, action sequencing, and error monitoring. These processes may become more fragile with ageing and may be further compromised in individuals with mild cognitive difficulties. Traditional manuals frequently increase this burden because users must translate written and often abstract instructions into concrete actions on a physical control panel. In contrast, contextual AR guidance is theoretically expected to reduce this translation effort by visually grounding each instruction in the real environment, narrowing the action space, and presenting procedures as manageable sequential steps. In this sense, the proposed system may be interpreted as a potential external cognitive support mechanism for instrumental daily activities, with possible implications for cognitive accessibility, perceived self-efficacy, and functional autonomy that remain to be empirically tested.

Accordingly, the present study is positioned as a technology-oriented proof of concept with relevance for cognitive accessibility, rather than as a clinical validation study in older adults with diagnosed neurocognitive disorders. The study is guided by the following research questions:

RQ1. Can a YOLOv8-based detection and segmentation pipeline identify and localize common appliance control elements, including knobs, push buttons, touch buttons, sliders, and toggles, with sufficient accuracy for real-time AR guidance?

RQ2. Can the proposed system architecture integrate appliance-control detection, manual-based language-model responses, tracking, and AR visualization into a coherent workflow for contextual step-by-step guidance?

RQ3. How do users perceive the preliminary usability, clarity, and acceptability of the AR-based guidance interface in a non-clinical proof-of-concept evaluation?

These research questions delimit the scope of the present work. Claims related to cognitive accessibility, cognitive workload, self-efficacy, and functional autonomy are therefore presented as theoretical implications and future research directions, rather than as outcomes directly demonstrated in the present study.

## 2. Related Work

In [4], the authors analyze why users do not like to use printed and digital manuals. Such kinds of studies find that manuals lack user usability since navigating through them is complex and contains many details. In another work [5], a video-based approach is used to improve the usability of traditional manuals. In this work, an AI-based approach is introduced to convert textual manuals into instructional videos. Based on their evaluation, 85% of users prefer to watch these instructional videos rather than read traditional manuals, strengthening the need for an innovative approach to following the manual and automatically extracting relevant information from manuals and presenting the result in an interactive and step-by-step manner to users. Another research [6] demonstrates the effectiveness of instructions with augmented reality compared to traditional paper-based user manuals in assembly training tasks. In this work, the authors evaluated the task completion time, error rates, workloads, and usability with the help of NASA-TLX [7] and System Usability Scale (SUS) [8] tests. They asked participants to assemble a planetary gearbox in three different situations: first with the help of paper instructions, second with an AR system based on HoloLens, and third with an AR system based on mobile devices. Results from this study show that instructions with AR reduce errors and improve usability.

Several existing studies are relevant to this work in different aspects, from augmented reality for user manuals, assistive technologies for users with impairment, and object detection for appliance buttons based on deep learning methods. Most of these studies are restricted to specific models or environments, lack real-time adaptability or require predefined appliances. One of the works in the Augmented Reality (AR) field discussed in previous research [9] introduced an AR-based interactive user manual built with Unreal Engine and HoloLens 2 to assist users in assembly tasks. To use this AR system, users can scan QR codes at designated stations to get fixed 3D models, images, and instructions adapted for real-world locations. In [10], an artificial intelligence-based module is proposed that gives real-time visual instructions for household appliances with augmented reality. This system was built with the help of classification, tracking, and matching algorithms, and finds the appliance using mobile-based classification models, gets the user manual, and finally overlays the guidance on the appliance interface. The system uses SuperPoint [11] and SuperGlue [12] networks, object tracking, and template matching. In this study, the system was based on a predefined template and model-specific appliance detection.

From the object detection perspective, which is a primary part of identifying the controls and buttons of the appliances, in [13], the authors featured a dataset and machine learning-based method for classifying house appliance buttons and visualizing the buttons on appliances in a way adapted for visually impaired people. They created a dataset of 13,702 images and trained the YOLOv5 model to detect the buttons on the appliances. To improve accessibility issues through augmented reality [14], focused on the accessibility challenges that visually impaired users have in interacting with appliances. Their approach emphasized haptic feedback, tactile buttons, and audio assistance to improve accessibility. The survey was conducted with 181 visually impaired participants, and a hands-on test was conducted with 12 selected users to identify the main barriers to the usability of appliances, including difficulties in recognizing interface elements, reliance on long-term memory, and lack of auditory feedback. Their study mentioned that high-contrast interfaces, large icons, and texts are essential for low-vision participants, and having clear visual and auditory feedback to confirm their interaction is crucial. In another study [15], the authors proposed a persona-based approach that identifies accessibility issues of elderly and disabled users in interacting with home appliances. They have provided a guideline for designing appliance interfaces suitable for a specific group of users. This study analyzes different user difficulties and provides a persona to guide design improvements. In [16], a blind assistance system was introduced that uses CNN, MobileNet V2, and YOLOv8 to detect home appliance buttons and panic buttons in public transport for visually impaired people to interact independently with these appliances. One of the significant uses of augmented reality is in industrial training and maintenance. The work in [17] provides real-time object recognition and step-by-step tutorials with the help of MobileNet-SSD v2. It offers an augmented reality smartphone application to electrical engineering students to facilitate their work with complex lab equipment. The system, which integrates computer vision techniques and 3D interactive models, gives them a real-time AR-based tutorial that facilitates the procedure for them. From the perspective of AI-based instruction generation and user manual parsing, with the improvement of large language models, there is a possibility of parsing user manuals and generating instructions in an automated way. In [18], the authors propose a graph-based artificial intelligence framework (TARA) to extract the structured information from the user manuals. This system helps the process of machine reading comprehension of user manuals and makes the situation easier for the artificial intelligence models to generate relevant responses.

The reviewed state-of-the-art concerns AR-based guidance systems, object detection for appliance interfaces, accessibility improvement for people with impairments, and parsing user manuals. However, these works are still fragmented or limited in scope. They lack a real-time, scalable solution that brings these components together.

## 3. Materials and Methods

This section describes the proposed system architecture and the main methodological components of the work, including dataset collection and analysis, object detection, instance segmentation, object tracking, attribute detection, instruction generation, ethics, privacy and data governance, and integration into the AR interface.

### 3.1. System Architecture

**Figure 1:**
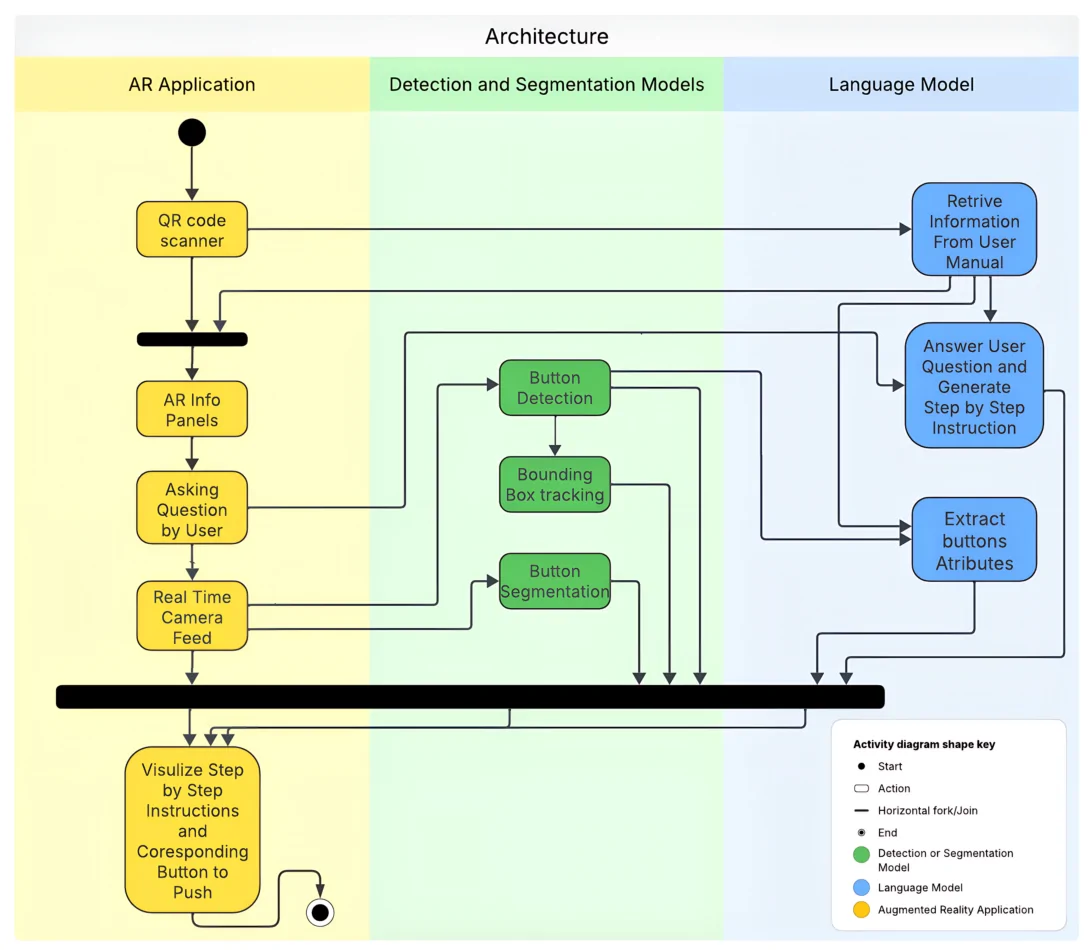
Overview of the proposed AR-based appliance guidance architecture, showing the interaction between the Unity mobile application, the computer-vision pipeline, and the Gemini language model.

As mentioned before, the primary goal of this system is to assist the user in using appliances and answer their questions through step-by-step guidance grounded in the appliance user manual and linked to the detected controls. This assistance is beneficial for users who do not have previous knowledge about the specific appliance, and reading the user manual is time-consuming for them. The system was also designed with potential accessibility-oriented applications in mind, although its use with visually impaired users was not directly evaluated in the present study. For this aim, the architecture shown in Figure 1 was designed to organize the interaction between the AR application, the computer-vision models, and the language model. Based on the proposed architecture, the system combines a computer-vision pipeline, a language model, and a Unity-based AR application.

As shown in Figure 1, the communication between the user and the application starts by scanning the QR code with the mobile application. The QR code is used to retrieve the corresponding appliance user manual, which is then provided to the Gemini 1.5 Pro API as manual-grounded context. The related information, containing the appliance model, useful tips, and energy-related tips, is shown in the application on the AR info panels. The user then asks their question, and the user’s query is sent to the Gemini API together with the retrieved manual context. Gemini prepares the user response by considering the user manual retrieved from the first steps, generates an answer based on the information available in the appliance manual, and gives a step-by-step guide to be shown in the application. In the next step, the real-time camera feed is sent to the detection, tracking, and segmentation models. YOLOv8 first detects the appliance buttons, which are then cropped from the original camera feed. BoT-SORT, as an object tracker, is used in this procedure to keep the identity and position of detected buttons stable across consecutive frames, so that the system can follow which control is relevant during each instruction step. Also, by using YOLO-Seg, the buttons available on the appliance are segmented on the original camera feed for real-time visual highlighting in the AR interface. The cropped button images and the appliance’s user manual are then sent to the Gemini API, and Gemini analyzes the cropped buttons to determine their color, shape, and relative position on the appliance. The extracted information is saved in a dictionary containing the detected button features and is used to support the matching between manual-based instructions and visible controls. In this step, sending the user manual is intended to ground the Gemini responses in the information available for the user’s specified appliance.

All the results, including the detected buttons with their bounding box and segmentation mask information, their attributes, and the answer to the user query, are sent back to the application. The Unity-based AR interface then visualizes the step-by-step instructions and highlights the corresponding detected controls on top of the real-time camera feed.

### 3.2. Dataset Collection and Analysis

For training an object detection model, the primary and important material is a high-quality dataset, enabling the object detection model to detect appliance buttons among different brands and environments. Due to the unavailability of a good and diverse publicly available dataset, a fully customized dataset was created in this work to ensure coverage of different appliance models in different conditions, but in real-world cases. However, it should be mentioned that there is another valuable published paper [13], which was done for creating a dataset of appliance buttons and developing an augmented interface. They have published their dataset, which contains images of appliances, but after analyzing their dataset, it was seen that now there are many new types of appliances that differ from what is collected in the published dataset, so there is a need to collect new images and make a new dataset for our project. The dataset created by us was diverse and ensured a reasonable basis for the training phase of the YOLOv8 model. The complete metadata file contained 13,081 image records, covering 24 appliance categories, 80 manufacturers or brands, 459 identifiable model names or numbers, and 462 unique appliance entries based on the combination of appliance type, manufacturer, and model. Among these records, 5575 images were annotated for object detection and included in the final annotated dataset used for model training and evaluation.

**Figure 2:**
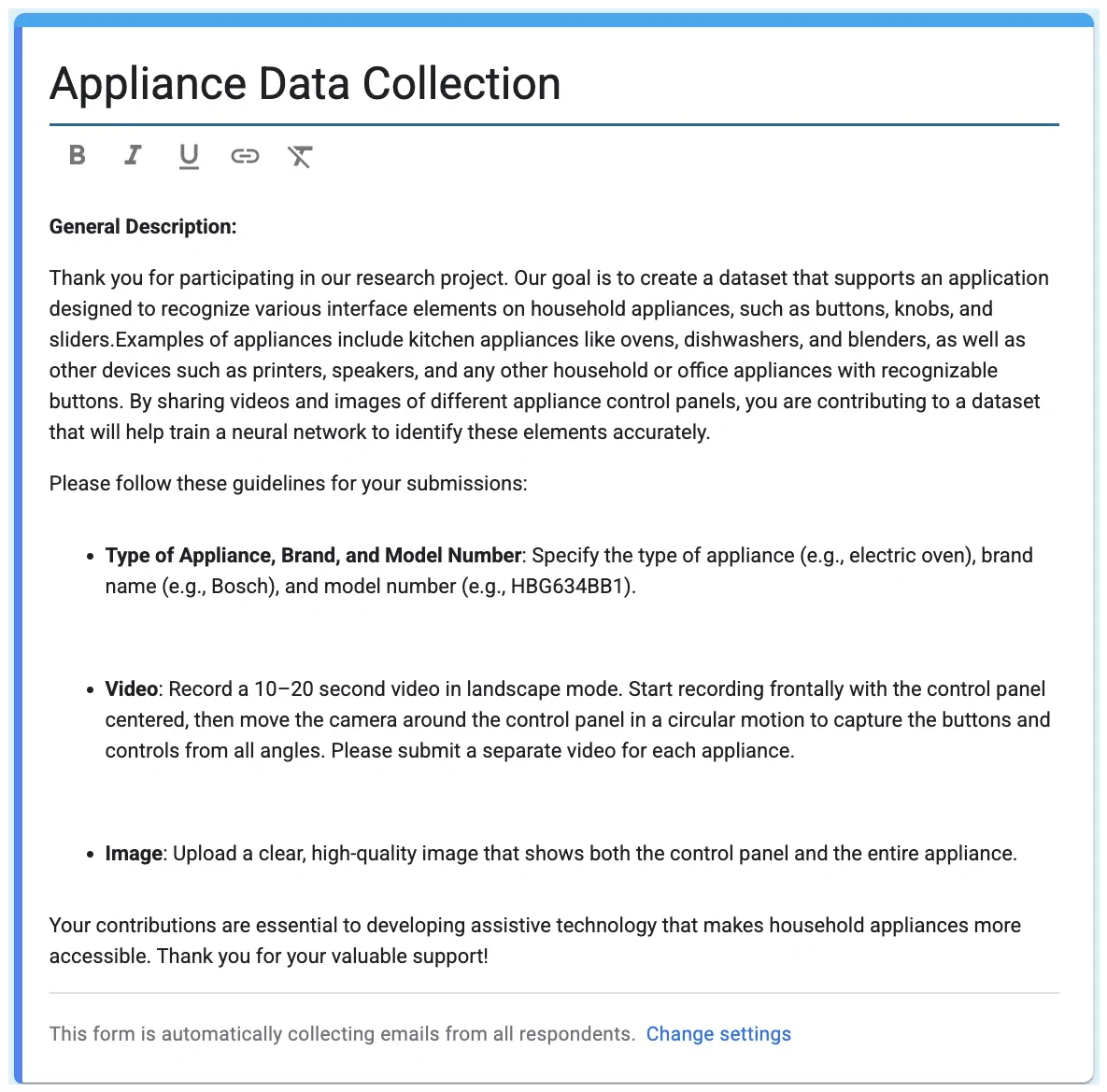
Google Form.

Three different resources were used to gather a high-quality dataset, ensuring that the dataset had a variety of appliances with different brands, button layouts, lighting conditions, and years of production, which contributed to a model that generalized better in the training and deployment phases. The primary source of images in the dataset is from appliances available in retail stores like MediaWorld, which is a retail store located in Rome. One single video from the appliance, one high-resolution image from the appliance, and one image from the label of the appliance that mentions the brand name and model number of the appliance were taken there. The video capturing is done through circular rotations around the appliance, to have a diverse perspective and angles of the appliance, since it is important for each button to be detectable while the user moves their mobile phone around the appliance in the augmented reality application. This approach helps to gather the dataset of appliances under different lighting conditions and angles, and includes the description of the appliance. A second source is images captured by real users. For this aim, a Google form was made and distributed among real users, in which they are asked to upload a video and an image of the appliances that they have in their homes or offices. They are also asked to mention the product brand and model if they are familiar with it. This approach adds an extra benefit to our dataset, adding real-world images in distinct home lighting and situations, and gathering images of appliances that are older than the models available in the retail stores. The form description is showed in Figure 2.

The last resource for dataset images is Amazon reviews. After analyzing the number of instances in each class in the dataset, an imbalance in the dataset was found in the class of appliances with sliders and toggles. To mitigate class imbalance, additional publicly available user-posted images from e-commerce review pages were collected and added to the underrepresented classes.

After gathering all the images and videos from different resources, this data should be processed and made ready for annotation. As mentioned earlier, instead of taking only one image from the appliances, a video is captured from an appliance in a way that, in a video, we have different angles and perspectives of the appliance by rotating the mobile in different directions. These captured videos should be converted to images for adding to the dataset, so different frames should be extracted from each video. For extracting the best frames from each video, different quality scores are evaluated for each frame to ensure that it is informative and also has a diversity of frames in the dataset. For this aim, the main criteria are sharpness, focus on the center, and lighting uniformity. Sharpness is computed using the variance of the Laplacian operator that detects the edges and measures how well they are defined. This way ensures that blurry frames are discarded. Another measurement is focused on the center, which is analyzed by extracting the middle portion of the frame by assuming most of the information is there, then Canny edge detection is applied on it, and the sum of the detected edges is the focus quality. The last criterion is lighting uniformity, which is determined by calculating the standard deviation of pixel intensities, helping to discard frames with excessive brightness variance in which the visibility of the appliance is not good. By summing the scores from these three criteria, a quality score is assigned to each frame, and the top 25 frames with the highest score are selected. To be sure about the diversity of frames, K-Means clustering is applied to these top-ranked frames and groups them based on their edge features into six clusters. From each of these six clusters, a representative frame is selected that ensures the variability in angles and lighting conditions, and there is no redundant frame in the dataset. Therefore, each source video contributed up to six selected frames to the annotated dataset, reducing near-duplicate frames while preserving variation in viewing angle and illumination. All of these steps are summarized in the Table 1.

A structured Excel file was prepared to manage the dataset and also prepare some facilities for additional research in the future. This file includes: image ID, appliance type to help categorize appliances for specific tasks, manufacturer and brand for brand-specific tasks, model number or name, and an annotation flag, which describes whether the image is already annotated or not. This structure enhances the dataset usability and monitoring annotation process for future training, and by documenting the manufacturer and model number, it can be used for tasks beyond button detection. With the inclusion of this metadata, the prepared dataset is not only suitable for YOLO object detection but also can be a robust foundation for projects in terms of classification application by type, model, or brand, and model recognition, and it can also be updated dynamically with new models in the future.

**Figure 3:**
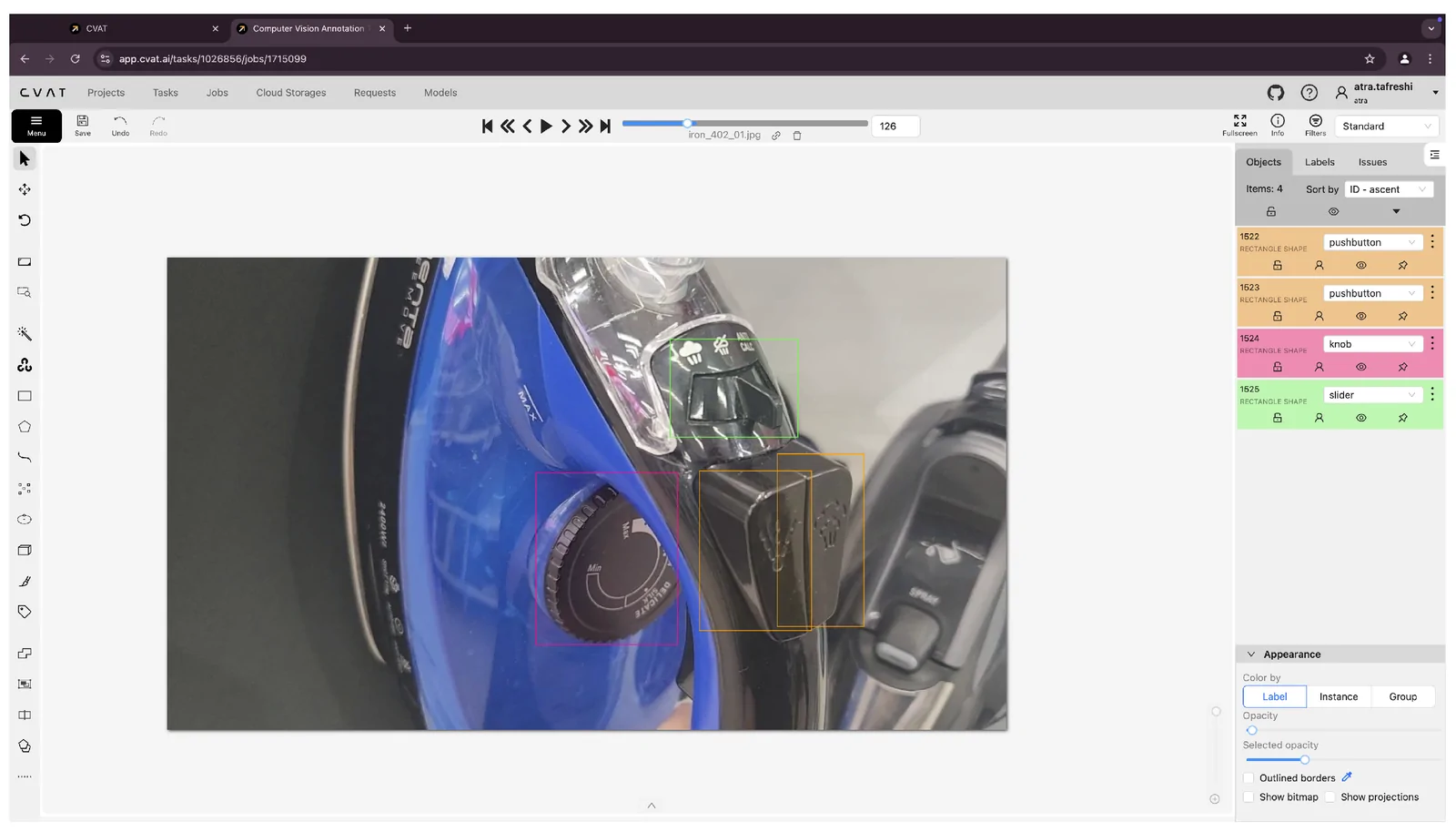
Manual annotation in Cvat. The colored bounding boxes represent manually annotated appliance control classes (e.g., push button, knob, and slider) used for object detection.

Another step that should be done before training the object detection model is dataset annotation. The dataset is annotated manually with the CVAT [19] platform, which is known as a free, user-friendly tool for labeling images. CVAT is a free, online, interactive video and image annotation tool for computer vision, and offers good support for people working in the academic field, and also supports drawing precise bounding boxes. It also supports collaborative annotation, and at the end, it is possible to export the annotation files in a format that YOLO supports for training mode. In our work, the six best frames were uploaded for the annotation process, and for each image, bounding boxes were drawn by hand around the specific buttons and labeled in 5 classes: knob, slider, push button, touch button, and toggle to ensure that the dataset is prepared for every button type. The bounding boxes were drawn as tightly as possible around the buttons. Annotation quality control was performed by the five researchers involved in the study, with particular attention to bounding-box tightness, class-label consistency, and ambiguous cases such as visually similar push-button and touch-button controls. Background elements and reflection were not included in the annotations to ensure that the system avoided introducing noise. The final annotations were exported to YOLO format to use in YOLO model training for button detection. The following Figure 3 shows the annotation procedure in the CVAT platform.

**Figure 4:**
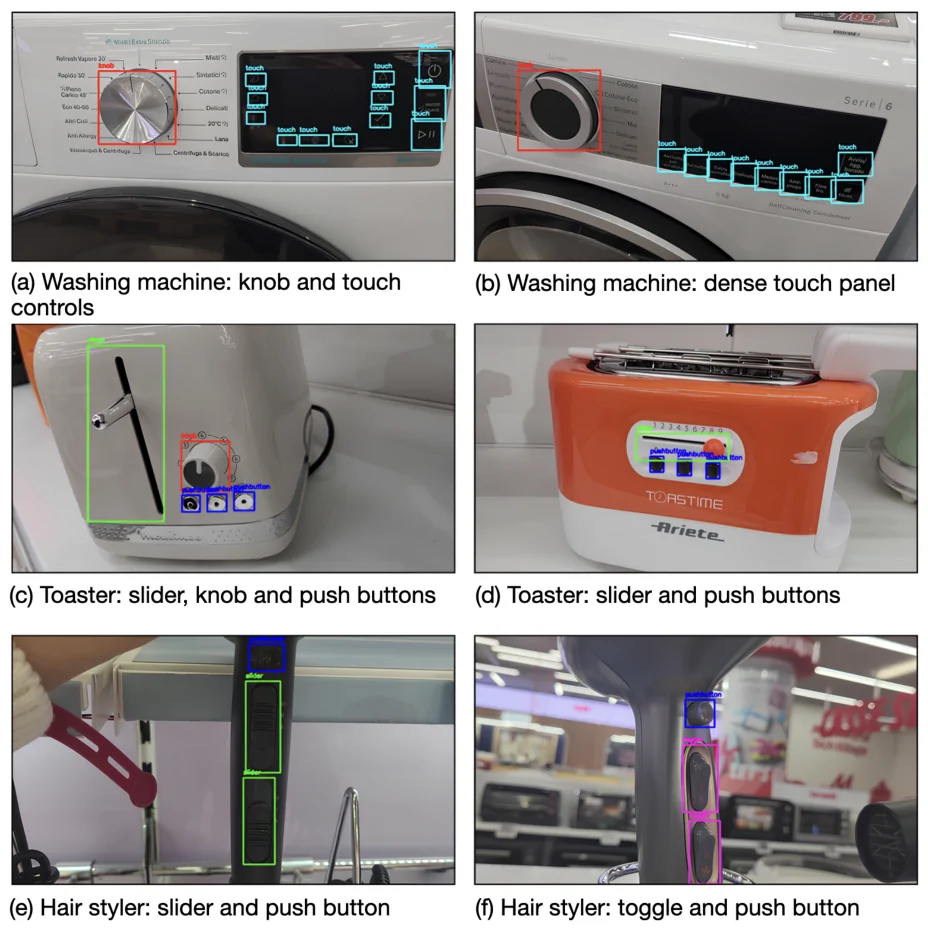
Representative annotated examples from the custom appliance-control dataset: (**a**,**b**) washing-machine interfaces with knobs and touch controls; (**c**,**d**) toaster interfaces with sliders, knobs, and push buttons; and (**e**,**f**) hair-styler controls with sliders, push buttons, and toggles. The examples illustrate different appliance categories, acquisition conditions, and control classes.

Representative annotated examples from different appliance categories and control layouts are provided in Figure 4 to illustrate the variability of appliance types, acquisition conditions, and control classes included in the dataset.

After analyzing the class distributions in our dataset, it was seen that some class distributions are imbalanced. The class-level distribution before and after adding supplementary slider images is reported in Table 2.

This table reports the class-balancing analysis used to document the effect of adding slider-focused images, while the final source-level annotation statistics are reported separately in Table 3. As shown in Table 2, push-button and touch-button annotations represented the largest classes, while sliders and toggles were less frequent. The imbalance was particularly relevant for sliders, since they are less common on household appliances and show higher variability in appearance. To compensate the limited number of slider examples, around 700 additional images were gathered from Amazon and eBay review photos to ensure the dataset has sufficient numbers of sliders for training the detection model. These e-commerce images were used only for research and model-training purposes, and redistribution of the corresponding raw images is subject to copyright and platform-policy restrictions. This addition increased the dataset size by 14.36% and led to a 121% increase in slider-class annotations. Although toggles and knobs were also less frequent than push and touch buttons, they were not further expanded because their shapes were more visually distinctive and more consistent across appliances. Furthermore, data augmentation techniques, including rotation, contrast adjustment, and brightness modification, were applied to increase the diversity of underrepresented classes in the training phase. Addressing these class imbalances improved the dataset’s representativeness, enhancing the model’s ability to detect and classify all button types more effectively. The final version of the dataset collected from different resources is reported in Table 3.

The final annotated dataset contained 38,199 annotations across 5575 images, corresponding to approximately 6.85 annotated control elements per image.

For model development, the annotated dataset was divided into training, validation, and test subsets using a 70%/20%/10% split, respectively. This split was used to evaluate the model on images that were not used during training. Future work will include a stricter external validation strategy, in which appliance models, source videos, and domestic environments are separated across training and testing to better assess generalization to unseen real-world conditions.

**Figure 5:**
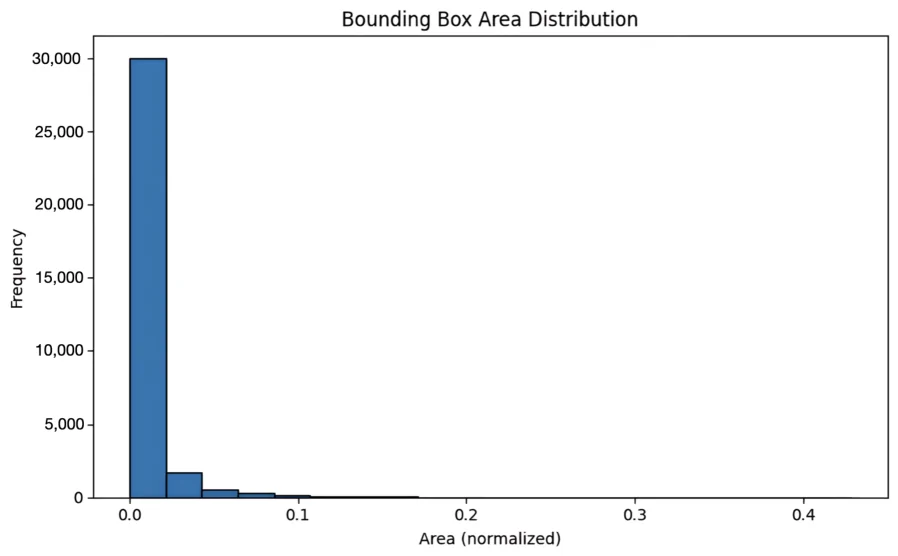
Bounding box area distribution.

About bounding box area distributions, as illustrated in the histogram of bounding box areas in Figure 5, most of the bounding boxes cover a small portion of the images as expected, since buttons of appliances in the real world are also a small proportion of the whole appliance. This small bounding box size makes the situation more complicated for object detection models to detect them.

### 3.3. Object Detection

Object detection is an important task in computer vision, as it aims to locate and classify objects in an image accurately and efficiently. In this research, the primary goal of object detection is to identify appliance control elements, including push buttons, touch buttons, knobs, sliders, and toggles. One of the main challenges in this task is the detection of small objects, because appliance buttons usually occupy only a small portion of the whole image. This can make feature extraction more difficult, especially when the controls are affected by limited pixel information, down-sampling in feature maps, occlusion, background clutter, reflections, or different lighting conditions. Models such as YOLOv8 [20] are suitable for this type of real-time detection task because they provide fast inference and support features such as anchor-free detection, dynamic input sizes, and improved feature aggregation.

The YOLO (You Only Look Once) family of models was considered because the proposed AR application requires real-time detection of appliance controls from camera input. YOLO models process the image in a single-stage detection pipeline and predict bounding boxes and object classes in one forward pass. This characteristic is important in this work because the detected buttons should be localized quickly enough to be used for real-time guidance and visualization.

Different YOLO versions were considered in this research, including YOLOv5, YOLOv8, and YOLOv11. YOLOv5 [21] was considered because it is widely used and computationally efficient, but in this task the main challenge was not only speed, but also the reliable detection of small control elements with different sizes and visual appearances. YOLOv11 was also considered as a newer Ultralytics model, but the larger model sizes require more computational resources, which was not suitable for the real-time requirements of this project. YOLOv8 was therefore selected because it provides a suitable balance between speed, accuracy, available model sizes, and compatibility with both detection and segmentation tasks.

The architecture of YOLOv8 is mainly similar to its predecessor, YOLOv5, but it includes several changes that are relevant to this work. Although there is no original peer-reviewed paper for YOLOv8, its official documentation describes the model and its implementation, and related studies such as [22] discuss the architectural changes and differences between recent YOLO versions. In this work, the most relevant characteristics of YOLOv8 were its anchor-free design, multi-scale feature representation, support for data augmentation, optimized inference, and native segmentation support. These characteristics are useful for appliance control detection because the target objects are small, visually diverse, and often appear in different lighting conditions and camera viewpoints.

Based on these considerations, YOLOv8 was chosen as the object detection model for this work. In [23], the authors reviewed the challenges and methods related to small object detection, showing that the choice of architecture and augmentation strategy has an important role in detecting small objects. This is relevant to the current task because appliance buttons are often small and can appear in dense control panels. In addition, ref. [16] reported the use of YOLOv8 for detecting small appliance-interface elements in assistive technology contexts. These works supported the choice of YOLOv8 for appliance button recognition in this study. Another benefit of YOLOv8 is its native support for segmentation through YOLOv8-Seg, which was used in this work to improve the visualization of detected buttons on the appliance interface.

After selecting YOLOv8 as an object detection model, the next step was to choose the model size, fine-tuning strategy, hyperparameters, and augmentation settings. YOLOv8 can be trained from scratch, or it can be fine-tuned using pre-trained weights or datasets related to the target task. Training and fine-tuning were necessary for having an optimized model for the specific object detection task. For this reason, two fine-tuning stages were used in this work.

As mentioned earlier, there is a published dataset related to this work, which contains images of appliance interfaces. Using this dataset and first fine-tuning the YOLOv8 on it was expected to improve the generalization of the model before training on the custom dataset. In the first stage, YOLOv8 was fine-tuned on a larger available dataset [13] for 150 epochs using the default YOLOv8 settings. In the second stage, the pre-trained model was fine-tuned on the custom-prepared dataset collected for this project for 120 epochs, using hyperparameters and augmentation settings more suitable for the small-object nature of the problem. This second fine-tuning helps the model to see a greater variety of buttons in different lighting conditions and environments. Also, it should be mentioned that for these two fine-tunings, different YOLOv8 model sizes were tested as described below.

Regarding model size, two versions of YOLOv8 were evaluated in this work: YOLOv8n and YOLOv8s. YOLOv8n is very lightweight and has a low latency in inference; these features make it suitable for cases when we need to deploy the model on edge devices. However, because YOLOv8n has fewer parameters than larger YOLOv8 models, it may have less capacity to extract detailed features from small buttons. The first try for training was done on YOLOv8n, which provided faster inference, but its learning capacity was not sufficient for this task. When this model was tested on appliance images, it gave a large number of false negatives, which means that the model could not detect some buttons correctly, and this reduced the reliability of the guidance pipeline. In this application, since the user should be guided through the instructions step by step, when some buttons are not detected, it would be a challenge to guide the user, and the system would not be usable in a proper way. On the other hand, when the model was tested on YOLOv8s in the second try, the results improved, especially in terms of detecting small buttons with higher confidence. Although YOLOv8s requires more computational resources than YOLOv8n, it provided a better balance between detection reliability and inference time for the scope of this work. In conclusion, after experiments, YOLOv8s was selected as the final object detection model for detecting the buttons, and the fine-tuning and deployment were done on this model.

For training YOLOv8s on different datasets, different settings were used. For the first fine-tuning of YOLOv8s on the external appliance-interface dataset, the model was trained using the YOLOv8 default settings. For the second fine-tuning stage, in which YOLO was adapted to the custom dataset created in this work, hyperparameters and augmentation strategies were selected with particular attention to the small-object nature of the problem.

As it was described for overcoming the issue of small object detection and also ensuring that the model will be better generalized in the future on unseen data, different augmentation techniques were investigated. Data augmentation was used to increase visual diversity and reduce overfitting to specific appliance layouts, lighting conditions, and camera viewpoints. One of the augmentation techniques used in this research is copy-paste augmentation, which duplicates objects from one part and pastes them onto another region of the same image or a different image. This method enhances the diversity of the photos, and according to a study conducted by [24], this augmentation can lead to a 7.1% improvement in small object detection performance. Another technique that should be emphasized is mosaic augmentation. In this method, four different images are combined in a single training image, and the model is introduced to objects in different backgrounds and lighting conditions to enhance generalization. Based on a recent study by [25], the mosaic augmentation method can improve small object detection, particularly in dense scenes, and it is beneficial for buttons that appear densely in control panels. Data augmentation parameters that were explored in this study, with their definitions, and justification for the button detection task, are provided in Table 4.

In addition, the batch size, learning-rate schedule, number of epochs, and optimizer were selected to balance training stability and performance. The default batch size of the YOLO, which is 16, was selected to optimize the usage of memory and prevent instability in training. While testing the different initial and final learning rates by adjusting them manually, it was seen that the default initial learning rate with the scheduler provided stable performance. Training was done for 120 epochs, as it was observed that the model continued learning up to this point, and after that, there were no significant changes in the model training. In the finetuning step, the chosen optimizer was AdamW, which allows for faster convergence, and since previously the training was done on a big dataset, now AdamW accelerates convergence by dynamically adjusting learning rates per parameter and makes it more efficient for fine-tuning.

Finally, although YOLOv8s provided the best trade-off among the tested models, some failure modes remain important for the proposed assistive guidance scenario. Touch buttons can be difficult to detect because they are often flat, low-contrast, symbol-based, or placed on glossy panels. False positives may occur when reflections, printed labels, icons, or background shapes resemble appliance controls. False negatives may occur under low lighting, motion blur, partial occlusion by the user’s hand, or when the button occupies a very small area of the image. For this reason, when the detection confidence is low, the system should not provide definitive guidance; instead, it should prompt the user to adjust the camera position, improve lighting, or confirm the intended control before continuing.

### 3.4. Instance Segmentation

Instance segmentation, also known as image segmentation, is the computer vision task of recognizing objects in images along with their associated shape. It is an extension of object detection, where each prediction includes a shape instead of just a bounding box defined by a center point, width, and height [26]. In this work, instance segmentation was not used as a separate recognition module, but as a visualization component to improve how detected appliance controls are displayed in the AR interface. When object detection is used alone, the application can only draw bounding boxes around the detected buttons. However, bounding boxes may be less intuitive for precise guidance, especially when several small controls are located close to each other on the same control panel. In these cases, instead of showing only bounding boxes, segmentation was used to display the detected controls more clearly and support button localization during the AR guidance process, particularly when the guidance step required highlighting a specific button or control element.

To keep the segmentation pipeline compatible with the detection model, YOLOv8-Seg was selected. YOLOv8-Seg extends the YOLOv8 framework to instance segmentation and allows pixel-level masks to be generated while maintaining the real-time characteristics required for the proposed application. There are several instance segmentation models, such as Mask R-CNN [27], Detectron2 [28], and DeepLabV3 [29]; however, YOLOv8-Seg was more suitable for this work because it is directly integrated with the YOLO framework used for detection, has lower computational cost than many two-stage segmentation models, and supports real-time processing.

For training YOLOv8-Seg, a segmentation dataset was required. Manual segmentation annotation is more time-consuming than bounding-box annotation because the object contours must be labelled at pixel level. To reduce this annotation burden, the Ultralytics auto-annotation function was used. This function uses an already trained YOLO object detection model to detect the target objects and then applies the SAM Model to generate segmentation masks. The resulting mask annotations were saved in YOLO segmentation format and used to fine-tune YOLOv8-Seg on the custom appliance-control dataset.

Segmentation performance may still be affected by small control size, glossy or reflective surfaces, low contrast between the button and the panel, partial occlusion by the user’s hand, and poor lighting conditions. These cases are especially relevant in domestic environments and are therefore considered in the limitations and future work of the system. Although the segmentation step increases the computational cost compared with detection alone, it was considered acceptable in this prototype because the masks provided clearer visual guidance for the AR interface.

### 3.5. Object Tracking

Based on the final goal of this work, which is showing detected buttons and step-by-step user-manual guidance in a real camera feed, an object tracker is essential to ensure that each detected button can be followed across consecutive frames. When the user moves the camera, the position of the appliance controls changes in the image. In addition, when the user interacts with a button, the control may be temporarily occluded by the hand or observed from a different angle. If detections are treated independently in each frame, the same physical button may receive different identities over time, which can create inconsistencies between the button highlighted in the AR view and the instruction generated for that button. Therefore, an object tracker was used to maintain a consistent identity for each detected control during the step-by-step guidance process.

Tracking small appliance buttons presents several challenges. Re-identification (ReID) is needed when a button is temporarily occluded and later becomes visible again, because the tracker should assign the same identity instead of creating a new track ID. Appearance similarity is another challenge, since many push buttons, touch buttons, and sliders have similar shapes, colors, and sizes on the same appliance panel. Real-time efficiency is also important because tracking must be performed while the camera feed, detection results, language-model outputs, and AR visualization are processed together. Several multi-object tracking algorithms were evaluated according to their ability to handle occlusion, ReID, and computational efficiency. A qualitative comparison of the considered trackers is provided in Table 5.

Various trackers were evaluated, and BoT-SORT (Bag of Tricks for SORT) [30] was selected because it provided a suitable balance between tracking stability and real-time applicability. BoT-SORT uses appearance-based matching to support re-identification, unlike simpler trackers such as Norfair [31] and ByteTrack [32], which rely more strongly on spatial association. Compared with DeepSORT [33], BoT-SORT also incorporates global motion compensation, which is useful when the mobile camera moves during interaction. FairMOT [34] can provide accurate tracking, but it is computationally heavier and was therefore less suitable for the real-time requirements of this prototype.

After YOLOv8 detects the appliance controls, BoT-SORT assigns and updates a track ID for each detected button across frames. This allows the system to associate each physical control with its corresponding detected region and guidance step, even when the camera position changes. BoT-SORT establishes correspondences between detections by combining motion prediction, spatial matching, and appearance-based re-identification. In the tracking pipeline, the Kalman filter is used to estimate the next state of each tracked object, while spatial association is performed using distance and overlap-based matching. Re-identification embeddings are then used to improve identity preservation when a control disappears temporarily and reappears after occlusion. This combination is useful for the proposed AR guidance scenario because the user may move the phone, change the viewing angle, or cover a button while pressing it.

The hyperparameters used for fine-tuning the BoT-SORT tracker and the justification for the hyperparameter choices are as follows:

- track-high-thresh (0.25): ensures only reliable detections are tracked;
- track-low-thresh (0.1): maintains tracking under lower visibility conditions;
- track-buffer (700 frames): preserves button identity during temporary occlusion;
- with-ReID (True): enables appearance-based identity preservation.

Despite these advantages, tracking remains sensitive to practical deployment conditions. Identity switches may still occur when several controls have very similar appearance, when a button is occluded for a long period, when the camera moves rapidly, or when lighting conditions reduce detection confidence. For this reason, future work should evaluate tracking stability under longer real domestic use, including cluttered backgrounds, hand occlusions, glossy surfaces, and different lighting conditions.

### 3.6. Attribute Detection

To present the user with guidance for using the appliances, it is necessary to highlight the buttons that should be used in each step of the instructions in a meaningful way. With the help of an object detection model, it is possible to draw the bounding boxes around the detected buttons on the appliance. However, telling the user the attributes of the buttons, like color, shape, and their relative position on the appliance, can help the user find the button more easily on the appliance. Also, whenever the object detection model fails in finding all the available buttons on the appliance, and it is not possible to draw a bounding box around the undetected button, the user can find the dedicated button used in the instruction, based on its attributes. This procedure of extracting the attributes of the buttons is a fundamental step for user guidance since, with the help of knowing these attributes, the user can simply find the desired button on the appliance. For having these features, several methods are investigated in this project, like using language models like Gemini [35], using Contrastive Language-Image Pretraining (CLIP) [36], Bootstrapped Language-Image Pretraining (BLIP) [37], and traditional computer vision techniques using OpenCV [38]. Among all of them, using Gemini for attribute detection with the help of the cropped button images and the user manual of the appliance is the selected approach in this project.

All these models have the ability to give us meaningful textual descriptions, but they are very different in what they generate and their reliability and accuracy in describing. It will be shown that models like CLIP and BLIP are not reliable for describing all these attributes of buttons, and also, the output of the OpenCV is not what is needed here and is very limited and simple, so the finalized approach is using the Gemini 1.5 Pro model as a button descriptor in our pipeline based on its powerful features. The multi-modal capability of the Gemini made a huge improvement in analyzing the images and texts of the user manuals at the same time. With the help of Gemini, the attributes of the buttons were extracted in a powerful way. In this section, the main focus is on the Gemini, its features, and its application in this work.

**Figure 6:**
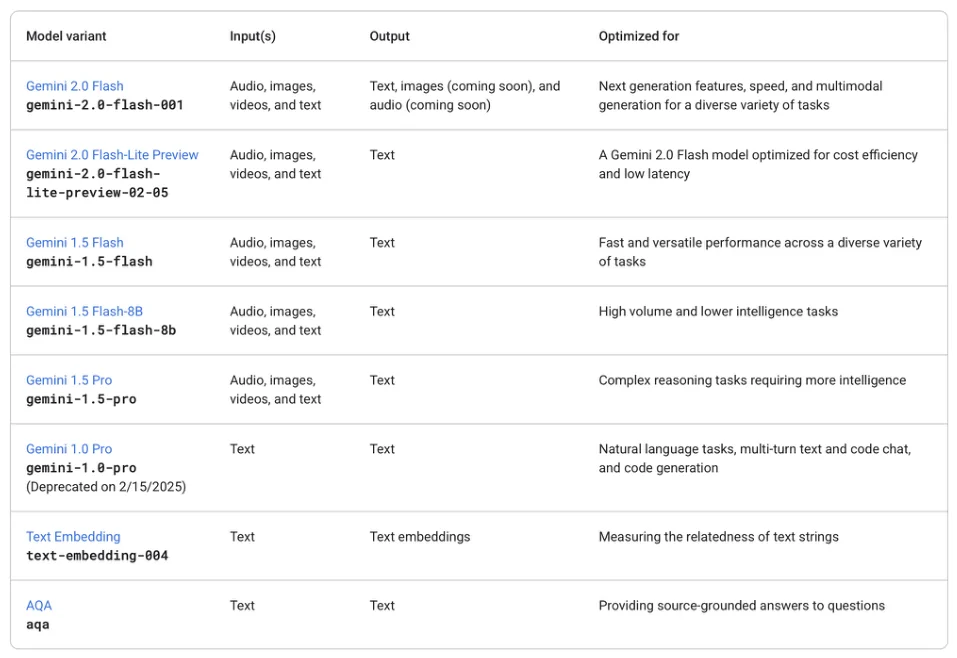
Available Gemini models.

Gemini is one of the advanced artificial models developed by Google DeepMind, designed to understand and respond to user queries, parsing and reading human-like texts, capable of doing multi-modal tasks. The previous models before Gemini are models like BERT [39], T5 [40], LaMDA [41], and PALM [42]. Gemini has followed the progress of these models and introduced improvements in efficiency and interpretability. Gemini is an advancement in the field of natural language processing and multi-modal artificial intelligence, and collects advancements from deep reinforcement learning, transformer architecture, and multi-modal data fusion. Gemini is available in different versions, and each of them is suited for different scenarios and can also be chosen based on computational resources. Figure 6 shows the detailed comparison between these models as mentioned on their official website:

These days, with an increasing improvement of language models, Gemini can be compared with the leading artificial intelligence models like GPT4 by OpenAI [43], Claude by Anthropic [44], and LAMA by Meta AI [45]. In the scope of this work, since the main focus is on using the language models in which their API is accessible, the focus is on comparing the GPT-4 and Gemini-1.5pro.

In this comparison, based on the work done in the paper [46] by the Gemini Team Google, it was seen that the Gemini 1.5 Pro achieves 100% recall of up to 530k tokens and >99.7% recall of up to 1M tokens. This task, while simple, provides a clear demonstration that Gemini 1.5 Pro is able to reliably retrieve information from long documents up to 1M tokens, while GPT-4 Turbo supported up to 128K sequence length by its API. Additionally, since Gemini is coming natively for multimodal tasks, in the paper mentioned above, it was demonstrated that Gemini is able to retrieve specific information across multiple hours of video. Gemini 1.5 pro can answer the questions on a video length of 10.5 h, while on the same task, GPT supports video length up to around the first 3 min.

Gemini models have many capabilities that make them suitable for a wide range of applications.

The first one is natural language understanding. Gemini is capable of understanding complex human queries and generating relevant responses. In this field, there has been a significant improvement in comparison to the traditional natural language processing models by involving deep contextual awareness, which allows it to retain the long-term dependencies along the conversation and also generate precise and human-like responses.

The second property of Gemini is multi-modal processing, which, despite earlier models that just processed texts as input, supports multi-modal inputs, including images, videos, audio, and texts. It can interpret the image and text at the same time and make it beneficial for applications where image-text correlation is needed. Gemini is capable of recognizing different objects in the images, answering queries based on visual and textual information, and also being helpful in tasks where the combination of these modalities is needed.

Another property that makes the Gemini advantageous is contextual adaptability. By this feature, with improved memory of Gemini, it is able to retain the context when there is a long interaction between Gemini and the user, and it does not lose the past information, giving the user an advanced experience in artificial intelligence applications.

Also, Gemini has broken down complex tasks step by step, like how human logic works; this process is named chain-of-thought (CoT) prompting, and because of this ability, it outperformed some models in the field of programming and math and making it suitable for artificial intelligence applications.

The final benefit of using Gemini is in its customization. Google provides an API and also the option for fine-tuning of the Gemini, so it can be adapted to different tasks and be applicable in many domains.

Since Gemini has multimodal capability, in this work, both the appliance user manual and the cropped image of the buttons were passed to Gemini for processing and getting meaningful descriptions from it. In this work, Gemini was asked to describe the button based on its color, shape, and relative position of the button on the appliance. Gemini showed a powerful ability to describe the buttons directly from the images. However, the power of the Gemini depends on the quality of the prompts that were passed to it and how this prompt is structured. In our work, there was a huge amount of work done on prompt engineering to optimize the Gemini outputs for describing the buttons in a desired way to be reliable and complete. Prompt engineering means that the input to the model should be designed and structured in a way that the model’s response becomes accurate and relevant to the query. Many attempts were made in this project to finalize the structure of the prompts passed to Gemini, since, with good prompt engineering, generating responses that are just guessing should be prohibited.

Accessing the Gemini model is done through its API, and since Gemini wants to maintain its system stable, it defines some rate limits for accessing the API. These limits are different in the service tier that is selected, but in this work, which is based on using the free tier of the Gemini, these limitations are very strict. For instance, in the free tier, the Gemini 1.5 pro allows users to send two requests per minute and 50 requests per day. So, a specific design, like putting some sleep parts in the code, is needed here to avoid problems on both the client and server sides. Not complying with these design strategies leads to errors, indicating the exhaustion of the resources. Before going to discuss in detail the applied approaches for button attribute extraction by the Gemini API, a configuration of the Gemini, used in this work, is summarized in the Table 6.

As seen from Table 6, a low temperature is preferred to ensure consistency and factual accuracy, especially when extracting button attributes and processing user manuals. This prevents misinterpretations. Also, a moderate value of 0.8 for top_p parameter ensures responses remain accurate while allowing diversity in how button descriptions and manual interpretations are extracted. Setting top_k = 64 balances accuracy and variation, ensuring slightly different but still valid responses while avoiding randomness. And finally, a high limit of 8192 tokens ensures that complex instructions, button descriptions, and user manual analysis are not prematurely cut off.

The approach discussed in this part is about using Gemini for attribute description based on the user manual and cropped images of the buttons. In this work, Gemini was not used as an unconstrained source of appliance knowledge. Instead, the user manual was kept as the main reference in the chat session, and the prompts were designed to ask for information related to the specific appliance and its detected controls. The power of this approach comes from detailed and investigated prompt engineering and post-processing of the Gemini responses. To reduce unsupported answers, Gemini was instructed to answer based on the information available in the user manual and the provided button image, rather than adding information from unrelated sources. Also, in this work, there is a solution for the cases where the YOLO object detection model fails to find all the buttons available on the appliance control panel. Although the goal of this project is to make the YOLO model as robust as possible, the detection models will not show us an accuracy of 100%, so in the Gemini part, there should be a recovery approach for this case. In this case, missing detections by YOLO are handled by querying Gemini about the buttons that were not detected and checking whether they are mentioned in the appliance user manual. If such buttons are described in the manual, Gemini is then asked to infer their attributes in the same way as for the buttons detected by YOLO. However, when the manual does not contain enough information, or when the button cannot be confidently matched with a detected control, the system should avoid giving a definitive button-specific instruction and should instead ask the user to confirm the control or adjust the camera view. Based on the sensitivity of the work when applied to future accessibility-oriented scenarios, the detected buttons and the instructions provided to users should be as accurate and reliable as possible.

**Figure 7:**
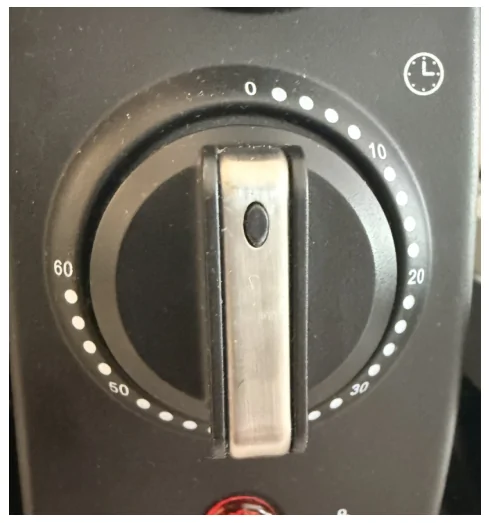
A cropped button of an appliance.

Describing attributes of buttons with the help of Gemini consists of three main steps as follows:

Step (1) Crop the detected buttons from the camera feed: In this step, when the Unity application captures real-time camera feed and send them through the websocket, one of the captured camera feeds in the first seconds is passed to the YOLO model for detecting the buttons on the appliance, and after that, the cropping function crops the detected buttons from the image based on their bounding boxes. This cropping function iterates through the buttons detected by the YOLO model, and for each button, it takes the center coordinates from the detection result. For cropping the buttons, the additional margin is defined to be sure that when the cropping is done, the buttons are in the cropped part, so the bounding box coordinates for cropping are calculated based on the original bounding box coordinates, plus a margin. Now, the cropped buttons are ready to send to the Gemini API for further analysis in the next step. A sample of a cropped button from a toaster is shown in Figure 7.

Step (2) Prompting Gemini to get the attributes of the buttons: Here, the cropped button was sent to Gemini, and it is asked to give detailed information about the different attributes of the buttons. The first attribute is the button name, for which Gemini will tell what the functionality of the button is based on the user manual. In other words, what is the name of this button in the user manual? The second attribute is relative position. Gemini is asked to say what the? position of the button on the appliance, with respect to other buttons on the appliance, and the final attribute is appearance, for which Gemini is asked to describe the appearance of the button, like what its shape, color, or any specific visual feature is. In this step, a new chat session is started that includes the user manual of the appliance and the uploaded, cropped image. This ensures that the Gemini will respond to the query based on the user manual and also know in detail what buttons we are talking about; this leads to an accurate response from Gemini. Although in the chat session, there are these references, a structured prompt should be sent to Gemini to get the relevant information. For the ease of further response processing, Gemini is restricted to a structured response, preventing it from adding extra information other than what is needed. In a chat session user manual of the appliance, and one of the cropped buttons are sent to Gemini as a reference, and then in the sent message to API, Gemini is asked to describe the specific button, and the prompt used for this aim is: “what is this button in this appliance based on user manual, also what is the relative position of this button on appliance? and what is this button look like?”. The important note is to limit the Gemini to answer in a specific format, ensuring that we get a response in a way that we want for future processing. This limitation was done through this prompt: “Write your response in this format: button: button name with no additional information, relative position: its relative position, appearance: what does this button look like?” The example response from Gemini will contain the name, position, and appearance of the button, based on what is written in the user manual of the appliance.

Step (3) Parse Gemini’s response and make a dictionary for cropped buttons: Whenever the response about the button details is ready, the post-processing is needed to extract the relevant parts from the response and add them to the dictionary, since in this work, the goal is to have a dictionary of all the buttons with their descriptions. As mentioned earlier, it was asked of Gemini to write its response in a way that includes some keywords, including button, relative position, and appearance, so whatever is written after these keywords is the description of buttons in these fields, and they will be added to the button dictionary. The response for each attribute should be parsed to extract the relevant parts from it, and finally, add them to the dictionary of buttons. Two other attributes, button class and the bounding boxes, are relevant and come from the YOLO detection result and are added to the dictionary for further use.

With the fact that the object detection model used in this work, like other trained object detection models, can not detect all the buttons on the appliance, there should be a proper design to resolve this issue. In real-world applications, when the user is guided with the instruction, assuming that there is a button mentioned in the instruction that YOLO did not detect it in the last steps, the problem is that the dictionary of the buttons containing their features which is constructed up to now do not have this button in it, because it was not detected by YOLO, so the button is not cropped from the captured image and was not sent to Gemini for attribute detection. For resolving this issue, first, all the buttons used in the instruction file are stored in the used buttons list, and all the buttons detected by YOLO, which are stored in the button dictionary, are saved in the detected button list. Now, whenever there is a button in the used button list that is not in the list of all buttons detected by YOLO, Gemini is asked to describe this button and add it to a separate dictionary for further use. By doing this procedure, there are also available attributes for undetected buttons, so by using these attributes, there is a possibility to describe the button for the user based on its color, shape, and relative position on the appliance. It is obvious that for this button, there is no information about its bounding box and class ID, but other attributes of the button are retrieved from the Gemini. For this aim, in contrast to detected buttons that there is a cropped image available for them, for buttons not detected by the YOLO, only the user manual is passed to Gemini and is asked through this prompt: “is {button_name} button mentioned in this user manual? If yes, what is the relative position of this button on the appliance? And what does this button look like? ”. If the button is mentioned in the user manual of the appliance, then in the response, we get the attributes of it, as done in the previous part.

### 3.7. Instruction Generation

Another crucial step in the pipeline of this work is generating comprehensive instructions for users. In this work, the goal is that users can ask their questions in natural language without determining any specific format to constrain them. People can ask different types of questions, from general questions about the appliance to specific needs like “How to melt cheese in this oven?” Or “How to use the self-clean of this iron?” The designed system is now capable of answering the specific needs of the user in a precise form. The first step for achieving this goal is having the user manual of the appliance, which can be obtained by scanning a QR code attached to the appliance that contains the link to the user manual.

Many approaches were tested in this work to extract the relevant parts from the user manual based on the user query. One of the investigated approaches was to use Facebook AI Similarity Search (FAIS) and Sentence Transformers to extract the relevant part of the user manual based on the user query; however, it was seen that this approach would not be as precise and detailed as what language models like Gemini can generate. Therefore, decided to use Gemini as a language model for answering user queries based on the user manual of the appliance. Gemini has the ability to get the user manual as a PDF file and then, based on the structured prompt sent by the system to it, respond in a detailed way to the user’s query. Since the generated instructions may concern appliances involving heat, electricity, water, or sharp components, the language-model response was treated as a safety-sensitive part of the system. Therefore, the intended behavior of the system is to ground appliance-operation answers in the retrieved user manual, avoid unsupported instructions, and flag uncertainty when the relevant information is not available in the manual. In such cases, the system should not create a new procedure from general knowledge, but should inform the user that the requested information cannot be confirmed from the available manual. Similarly, when the detected button cannot be reliably matched to the instruction, the system should ask the user to confirm the control or reposition the camera before continuing.

**Figure 8:**
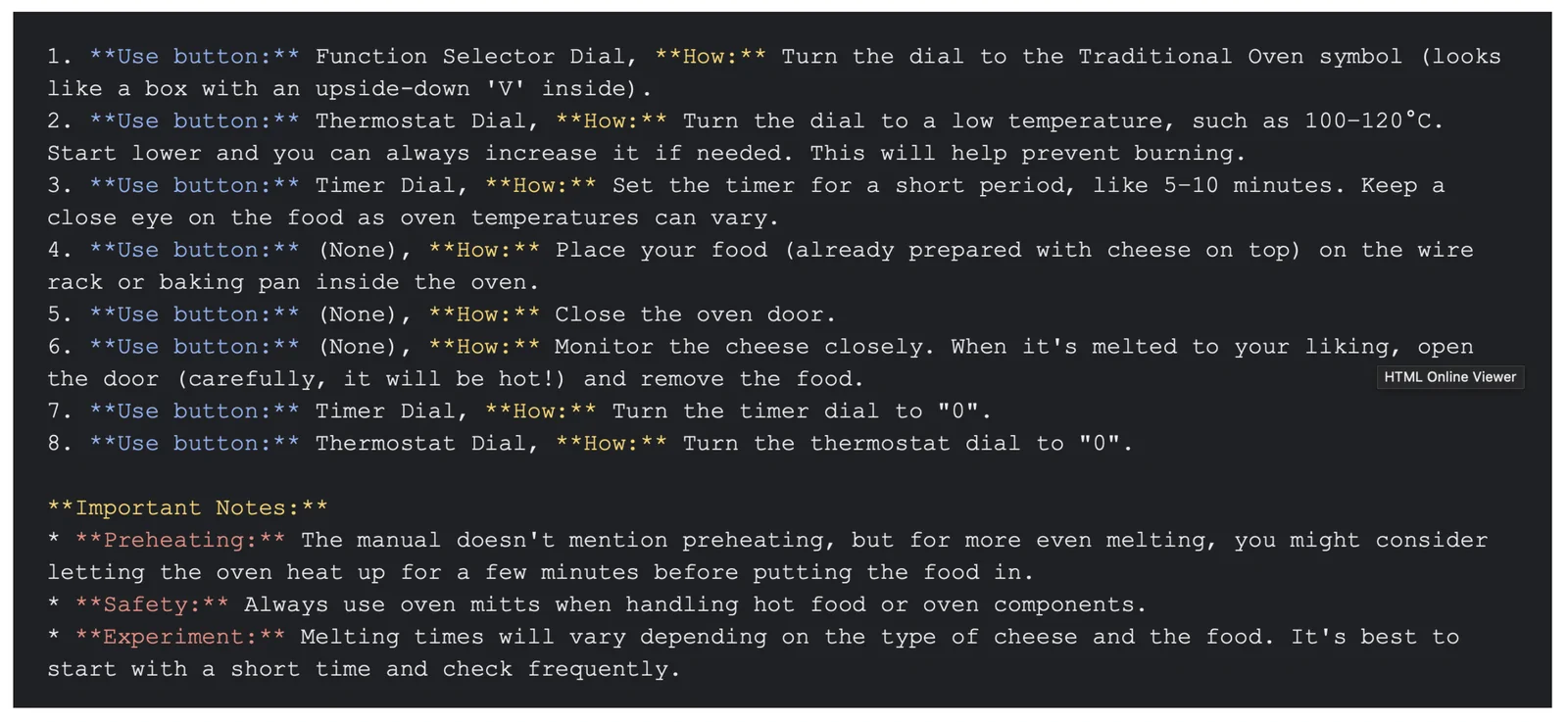
Original Gemini response before post-processing. The asterisks are part of Gemini’s original Markdown formatting.

When the users submit their query, there should be an approach to make step-by-step instructions for users to guide them through their needs. In this scenario, the user query, along with the appliance user manual, will be sent to Gemini, and it is responsible for responding in a structured way. Then, there is a post-processing step that cleans up the Gemini response and makes the instruction ready for the user. Two steps are done in this procedure:

Step (1) Send the user query and user manual to Gemini: First, the chat session with Gemini will start, containing the user manual. This ensures that the generated instruction will be fully based on the user manual of the specific model, and they are not coming from elsewhere. After analyzing this document, Gemini now has an understanding of the appliance and will give the responses for the specific appliance model. Then, a structured prompt containing the user query will be sent to Gemini and ask it to respond in a clear and step-by-step format, containing the steps that should be done by the user and the relevant button in each step. The sent prompt to Gemini is: “{user_query}, tell me what I should do step by step. Write your response in a way that in each line you tell me which button I should use on the appliance ”. The {user_query} is referring to the question of the user. The response from Gemini will be in a format in which, in each step, it is clear which buttons should be used and how. So, users can follow the generated instruction more easily on the real appliance interface. To reduce unsupported or unsafe responses, the language-model component was controlled through backend instructions. The backend provided Gemini with the retrieved appliance manual, the user query, and the detected button information. The prompt instructed the model to separate general task information, such as recipe-related steps, from appliance-specific operation. Appliance-specific settings, available functions, button usage, temperature selection, and safety-related instructions were required to be based on the retrieved manual. When the required appliance-specific information was not available or was uncertain, the model was instructed not to invent unsupported steps and to ask the user to check the manual or confirm the setting before continuing. This design was intended to reduce hallucinated appliance-operation guidance, especially for appliances involving heat, electricity, water, or other safety-sensitive functions.The sample of the Gemini response is shown in Figure 8.

Step (2) Preparing the instructions for the user: Once Gemini responds, its response will be saved in a text file and will be ready for post-processing that is needed. Since Gemini’s response includes the characters and additional information, the response should be cleaned, and for further needs, each step should be distinguished from the other steps in the instruction file and saved in a list of instructions. Finally, this list contains the steps that the user should follow to resolve their need.

### 3.8. Ethics, Privacy, and Data Governance

For ethical consideration, the consent agreement was included in the Google Form, in which people were informed that their submitted data would be used only for academic research purposes, including publications and scientific studies. It was also mentioned that all the data would be stored securely and that access would be restricted to authorized research team members only. No personal information, such as names or contact details, was shared publicly. It was also declared that this study adhered to data protection laws, including GDPR. Measures were taken to anonymize personally identifiable data wherever possible. By using these ethical considerations, the dataset collection process maintained transparency and ensured voluntary participation.

In addition to these measures, only appliance-related images and videos were requested from users, and the collected material was used for the technical development and evaluation of the prototype. When user-submitted images contained potentially identifying elements, such as personal objects, faces, addresses, or other private background details, these elements were removed, avoided, or anonymized where possible before further use. Access to the raw data was limited to the research team, and the dataset was not publicly redistributed in its raw form.

For the customer review images obtained from Amazon, permission to use review photos for research purposes was obtained by email from Amazon. These images were used only for academic research and model development, particularly to improve the representation of underrepresented button classes. Nevertheless, because such images may still involve platform-specific reuse conditions and user-generated content, any public sharing of the dataset will be limited to material that is permission-compatible, anonymized, or replaced by representative or synthetic examples when needed.

The dataset will be made available from the corresponding author upon reasonable request after publication, subject to privacy, copyright, permission, and data-protection restrictions.

The prototype also uses cloud-based language-model processing for user queries, appliance manuals, and cropped button information. This raises privacy and data-transfer considerations, especially for future use with older adults or users with cognitive impairment. In the present prototype, the transmitted information was limited to the data required for generating appliance-specific guidance. Future deployment should further investigate privacy-preserving strategies, such as minimizing the transmitted data, processing sensitive information on a secure server, avoiding the transfer of identifiable household information, and informing users clearly about how their data are processed.

The current study did not involve clinical intervention or the collection of medical or diagnostic information. The user evaluation was conducted as a preliminary usability assessment with non-clinical participants. Future studies involving older adults, visually impaired users, people with mild cognitive impairment, or other potentially vulnerable populations should include formal institutional ethics approval when required, appropriate informed-consent procedures, accessibility accommodations, and additional safeguards for data protection and participant autonomy.

### 3.9. Integration into the AR Interface

The last stage of the implementation is the development of an augmented reality application with Unity, which is a cross-platform game engine that supports both iOS and Android for mobile application development. The AR application contains four different scenes that are designed to assist the user in interacting with the application. These scenes will be described in detail, including the technical implementations and network communications. The scenes include QR Code Scanning and Data Retrieval, AR Visualization of Appliance Details, User Query, and button detection and guidance. To achieve this for exchanging data between the user interface and the deep learning models, WebSocket communications were employed to exchange data in real-time, and in this case, the heavy computations of the detection, segmentation tracker, and language model were on the server side rather than directly on the user’s mobile device. While some lightweight machine learning libraries are available on Unity for running AI inference directly on the mobile device, such as Lightship [47], Sentis [48], and Barracuda [49], because of their lack of the capability to integrate all functionalities required of this project, they are not used. Therefore, the task can be performed in real time, and the user’s mobile device does not need to have a specific configuration while the heavy computations are done on the server.

For data exchange between the Unity application and the AI server, multiple communication protocols, including Flask (HTTP Requests), UDP Socket Networking, WebRTC, and WebSockets, were considered. Finally, the web socket was ultimately selected based on its features, which are well-suited for this research for real-time interaction and bidirectional data flow.

The AR application is designed based on user-friendly interaction and accessibility. Each scene starts with textual guidance to have clarity and enable seamless user interaction. For better understanding, the screenshots of each scene and its dedicated technical details are provided in the following subsections. In the whole application, Design choices are based on accessibility. Therefore, Large fonts and high contrast are chosen to ensure readability for users of varying visual abilities. Also considered the minimal cognitive load; thus, instructions are kept short, precise, and step-by-step to avoid overwhelming users.

#### 3.9.1. Scene 1: QR Code Scanning and Data Retrieval

The first scene of the application starts with a welcoming interface and clear instructions for beginning the QR code scanning process. The design is focused on simplicity to make each step straightforward for all users. Additionally, a QR code placeholder is designed to facilitate the process of QR code scanning.

A QR code scanning mechanism was integrated within the Unity-based AR system to establish an efficient and structured method for retrieving appliance-specific user manuals. This mechanism allows downloading the appliance user manual by scanning a pre-generated QR code, which contains a unique URL that directs the system to the corresponding user manual (PDF). Once the QR code is scanned with the user’s mobile device, the retrieved URL is transmitted to the server with a WebSocket protocol, where the user manual is downloaded and processed to extract device-specific information. The language model then leverages the extracted details to provide step-by-step interaction guidance for the user. The ZXing (Zebra Crossing) library [50] was used to ensure accurate QR code detection. ZXing is an open-source barcode processing library that is used for scanning multiple barcode formats, including QR codes. The barcodeReader Decode() method processes frames captured by the device camera, then extracts the QR code, and decodes the embedded URL. The extracted QR code URL is temporarily stored in a local variable (lastResult) before transmission to the WebSocket server. The TextMeshProUGUI component was used to display real-time status messages to provide user feedback and improve interaction transparency. These messages indicate the detection of a QR code and the transmission of data to the server. Once the transmission is complete, display “Done!” And the scene transition starts.

The user manual is downloaded and stored. Once the QR code is received by the Python 3.11.9 server, the manual is stored for further processing by the language model. The user manual is analyzed and sends back the related info, context-aware guidance to the Unity application (Unity 6) in the next scene. In conclusion, the first scene consists of these elements: a Welcome Message, Instructional Guidance, an Interactive Button, a QR Code Placeholder, a Scan Button, and a Feedback Mechanism to ensure that the scanning process is done.

**Figure 9:**
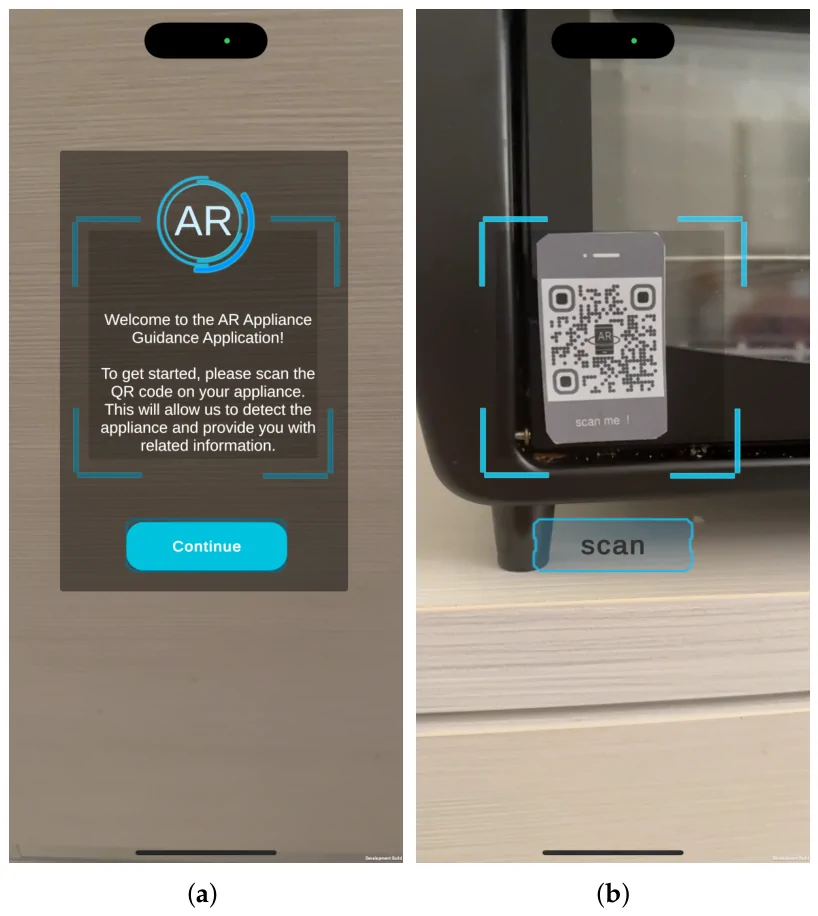
Scene 1: Application Welcome Screen and QR Code Scanning. (**a**) The welcome screen introduces the user. (**b**) User scans a QR code to begin.

Figure 9a,b illustrate the transition from the welcoming screen to the scanning phase, focusing on the placeholder and user-driven interaction through the “Scan” button.

An alternative approach was considered besides implementing a QR code-based solution to retrieve the appliance user manual. The initial strategy was based on image and model recognition to extract the appliance brand and model and then search for the related user manual in PDF format. However, due to the visual similarity between many appliance models and the difficulty of recognizing exact model numbers from images, the QR-based approach was selected because it provides a more reliable link to the correct manual. At the same time, this choice also introduces a limitation. QR code scanning may create usability barriers for users with motor, visual, or cognitive difficulties, and it also requires a sticker or marker to be available on the appliance. Future versions of the system could consider complementary entry methods, such as appliance image recognition, barcode or model-number recognition, NFC tags, voice-assisted manual selection, or caregiver-assisted setup.

#### 3.9.2. Scene 2: Visualization of Augmented Reality Panels

In the second scene, the AR panels are generated automatically around the QR marker in the real-world environment around the appliance. These panels are automatically filled with information for the following purposes:

- Video Panel: Displays multimedia content such as recipe suggestions, appliance usage instructions, or maintenance tips tailored to the detected appliance.
- Energy Tip Panel: Provides recommendations on energy-efficient usage. For example, suggesting users unplug appliances when not in use to reduce standby power consumption.
- General Tip Panel: Offers broader guidance on optimal appliance usage or care, enhancing usability and longevity.
- Appliance Identification Panel: Presents the appliance’s name and model number, allowing users to confirm the system’s recognition before continuing.

**Figure 10:**
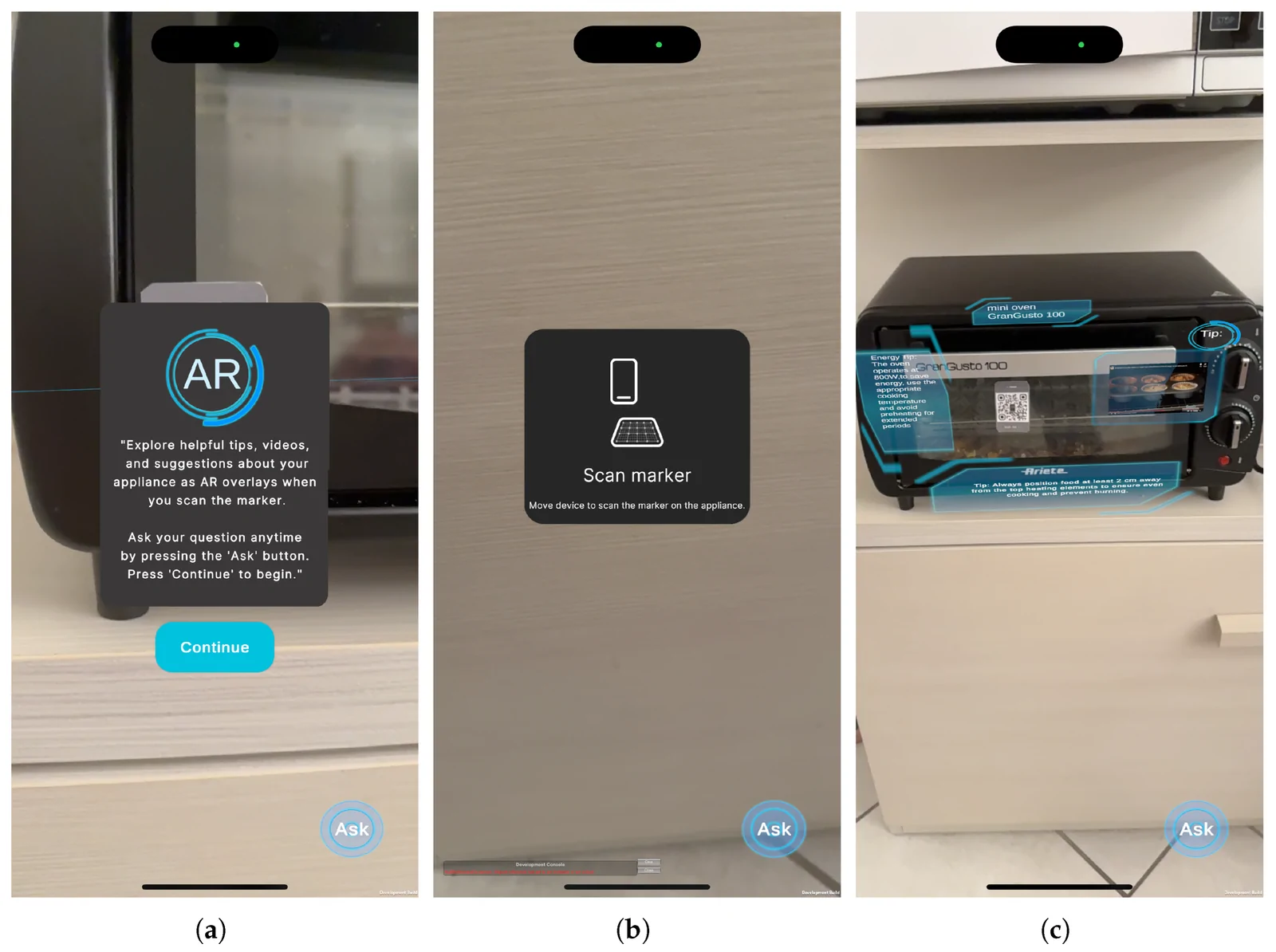
Scene 2: (**a**) Initial Guidance Panel. (**b**) Guide the user to Scan the Marker. (**c**) Displaying AR panels and information.

As shown in Figure 10, the second scene starts with a short guidance and then asks users to scan the marker and finally displays the AR panels around the marker on the appliance.

The core function of this system includes marker tracking and placing AR elements, web socket communication for information retrieval, and dynamic information that comes from a language model based on the user manual.

**Figure 11:**
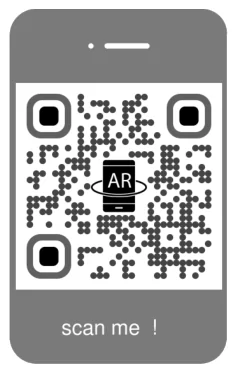
Dual-purpose design QR code marker.

The system utilizes ARTrackedImageManager to track the marker and visualize the AR component in the correct position in the real environment with the mobile device camera. This is a key component in AR Foundation, which is a library in Unity that detects and tracks predefined images. When a specific image (such as a marker or logo) is recognized, the AR system instantiates an interactive UI panel around the marker’s position. The tracked marker should be predefined in Unity. To reduce the need for additional stickers, the QR code itself is used as the tracking marker for AR placement. However, the content of the QR code itself is not relevant for tracking purposes; instead, Unity captures its frame and a central image as the predefined reference for tracking. Therefore, the combined sticker serves as both a QR code for accessing the raw user manual in PDF format and a marker for tracking and displaying AR overlays around the appliance in the real world. This is achieved by combining a center image and frame with a QR code shown in Figure 11. This ensures that the marker functions consistently, regardless of the specific QR code that is inside. On the other hand, since the QR code content varies, it can be used as the primary goal for user manual scanning. Additionally, it can be used independently of the AR application to access the user manual in its raw PDF format. This provides users with the flexibility to refer to the manual anytime, even outside the AR environment. There are two main ways to generate stickers. One is User-Generated Stickers; Users can easily create stickers for any appliance by linking a specific QR code to the manual, applying the provided marker design, and printing and sticking it to the desired appliance. This approach ensures accessibility for existing appliances that were not originally equipped with AR-enabled user manuals. Another way is manufacturer adoption; In the future, manufacturers can pre-install these stickers on appliances, providing users with AR-enabled manuals that are directly out of the box. This creates a seamless user experience while reducing the need to print user manuals.

After the marker is detected, Unity instantiates the related prefab and aligns the appliance-information panels with the real-world marker position. In this prototype, the prefab contains the AR panels used to display appliance details, manual-based tips, and the related video content. These panels are dynamically updated with the data retrieved from the server.

#### 3.9.3. Scene 3: Query Submission

**Figure 12:**
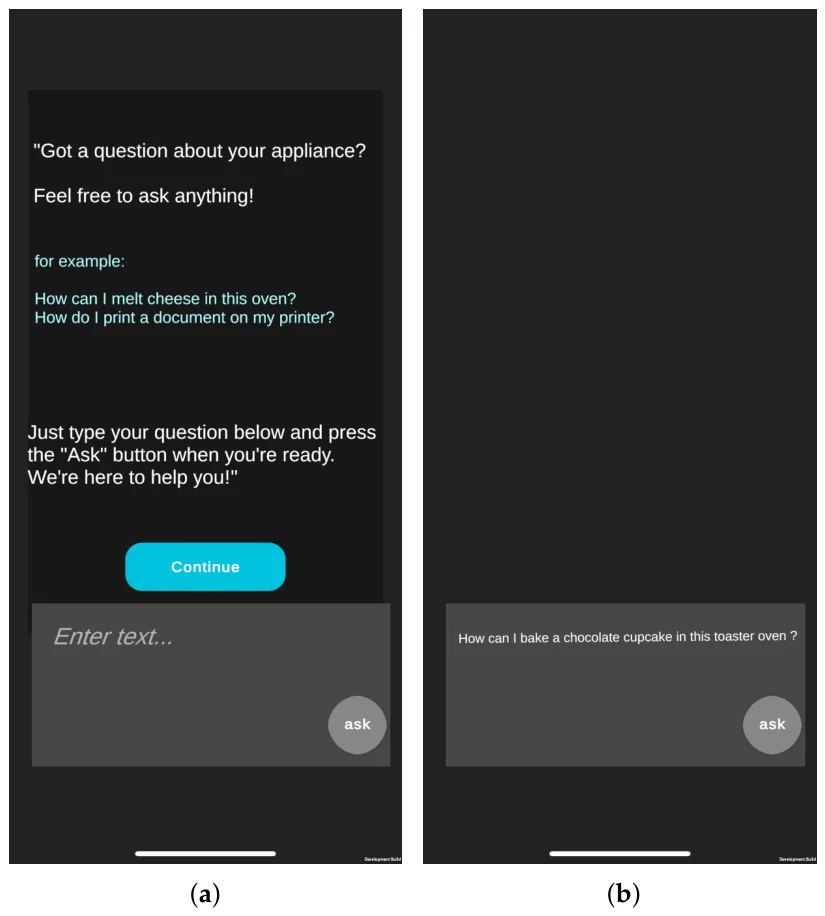
Scene 3: (**a**) Initial guidance panel. (**b**) Question input field.

A query submission mechanism has been developed to enable a more natural and adaptable user interaction, allowing individuals to ask appliance-related questions through either text input or voice commands by using their mobile phone’s voice-to-text functionality to enhance accessibility. This scene is activated after the user presses the “Ask” button in Scene 2. It is designed to give users a text box to ask specific questions about their appliance, as illustrated in Figure 12. This system is structured to handle a diverse array of inquiries, such as:

- Cooking Instructions—for example, “What are the steps to bake a cheesecake using this oven?”;
- Maintenance and Cleaning Guidelines—for instance, “What is the proper method for cleaning a coffee machine?”;
- Troubleshooting Assistance—such as, “What does a red light error message on my printer indicate?”;
- General Appliance Operation—for example, “How can I print a double-sided document?”.

Rather than requiring an instant response, the query is submitted, and the user moves forward to the subsequent scene, and the question is sent with the WebSocket to the server.

#### 3.9.4. Scene 4: Real-Time Object Detection and Guiding Instructions

The last scene is the main scene for real-time button detection and user guidance, and it is designed to assist users step by step in real-time. In this scene, the camera captures the actual feed and the corresponding button to push, highlighted with some visual elements or even segmentation masks, and also tracked at the same time in the whole process. Additionally, in a text box, the language model guides the user step by step based on the user’s previous question. Highlighting the button to push and related textual guidance by the language model are aligned together and visualized at the same time.

The real-time guided interaction system operates through a structured sequence of processes to ensure seamless user interaction. The ARCameraManager is responsible for capturing frame-by-frame input from the user’s mobile camera, continuously streaming data to support real-time functionality. Each frame undergoes pre-processing, including rotation and formatting, before being transmitted in a compatible structure. Once prepared, the frame is encoded in JPEG format and sent to the server with WebSocket, which facilitates low-latency, bidirectional communication between Unity and the AI backend. Upon receiving the frame, the server processes it using the Custom YOLO object detection model to identify button locations and their corresponding class. Instead of relying solely on bounding boxes or segmentation masks, the system selects the most suitable visual element to highlight the relevant button. To maintain consistency in tracking detected buttons throughout the interaction, unique track IDs are assigned. The detected objects are then matched with the system’s step-by-step guidance, ensuring that the appropriate button is visually indicated. The processed image, now featuring the necessary visual overlays, is sent back to Unity, where it is displayed in real time. As the user interacts with the system, the corresponding instructional text is provided alongside the detection results.This structured process was designed to provide step-by-step visual assistance, although its robustness under all real domestic conditions still requires further evaluation.

The scene begins with a panel displaying instructions on positioning the camera for a better object detection experience. Users are advised to maintain a distance of approximately 50 cm and point the camera toward the appliance control panel. This instruction was added because camera distance, viewing angle, lighting, reflections, and partial occlusions can affect detection quality in real use.

**Figure 13:**
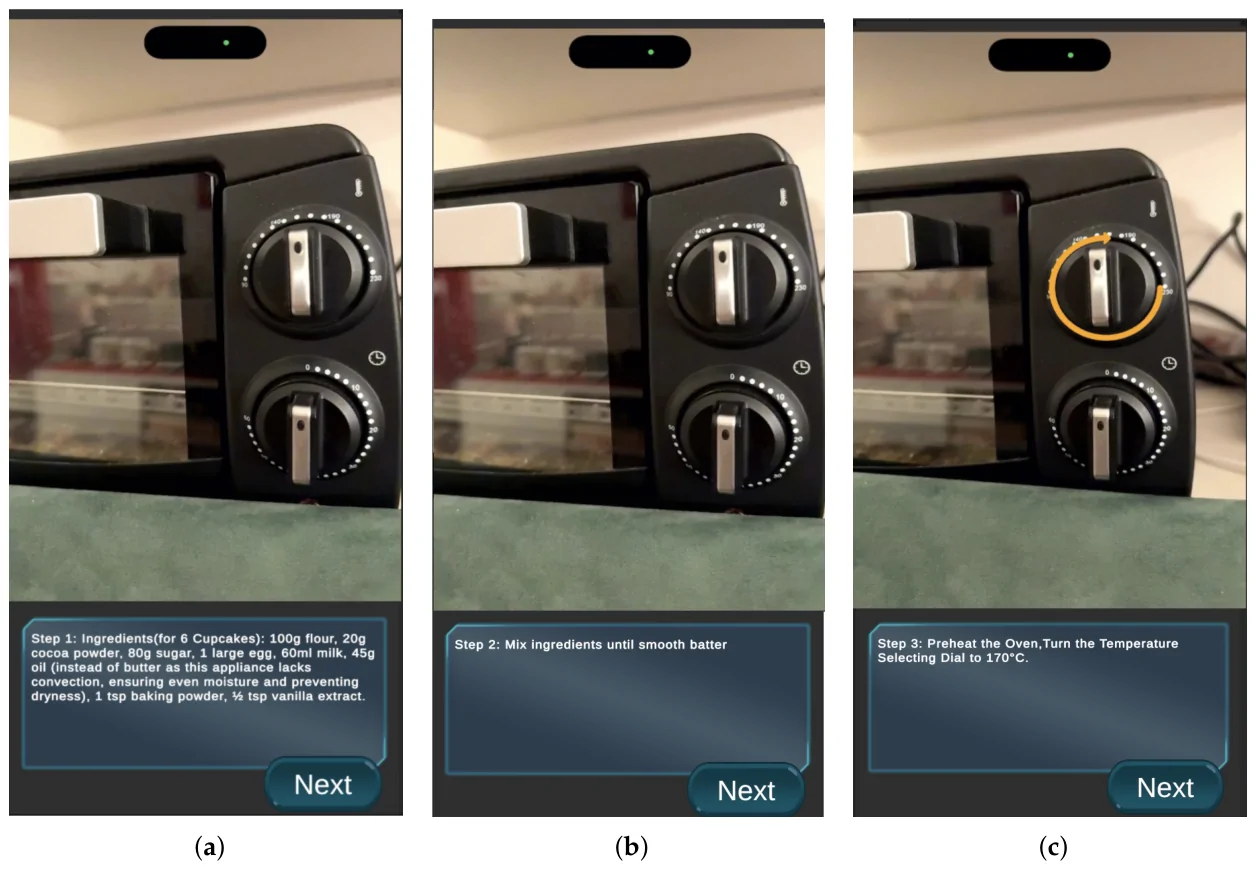
Scene 4: (**a**) Cupcake ingredients. (**b**) Preparing guidance. (**c**) Guide to turn dial.

Live Camera Feed is a window that displays the appliance in its current state. The AR system highlights the relevant buttons for each step based on the detections and instructions. For example:

A knob that needs to be turned is surrounded by a round arrow to indicate rotation. shown in Figure 13c.

A button for pressing is highlighted using a segmentation mask or bounding box.

The panel at the bottom provides textual guidance for each step with typing animation. This text dynamically updates as the user progresses through the task. Additionally, it is customized to the user’s query and answers the user’s question based on the appliance’s user manual. The figure illustrates this feature. To enhance user experience and avoid confusion, Buttons are highlighted only when relevant to the current step and instructions. For example, during the preparation of the ingredients, no buttons are highlighted as shown in the Figure 13a,b. Furthermore, the tracking system was used to keep the highlighted control consistent when the user moves the camera, interacts with the appliance, or slightly changes the camera angle. However, strong occlusion, rapid movement, or poor lighting can still affect tracking stability. By pressing the next button, users can complete each step and proceed to the next.

#### 3.9.5. Application Design Choices and Customizable Color Palettes for Accessibility Color Blindness

**Figure 14:**
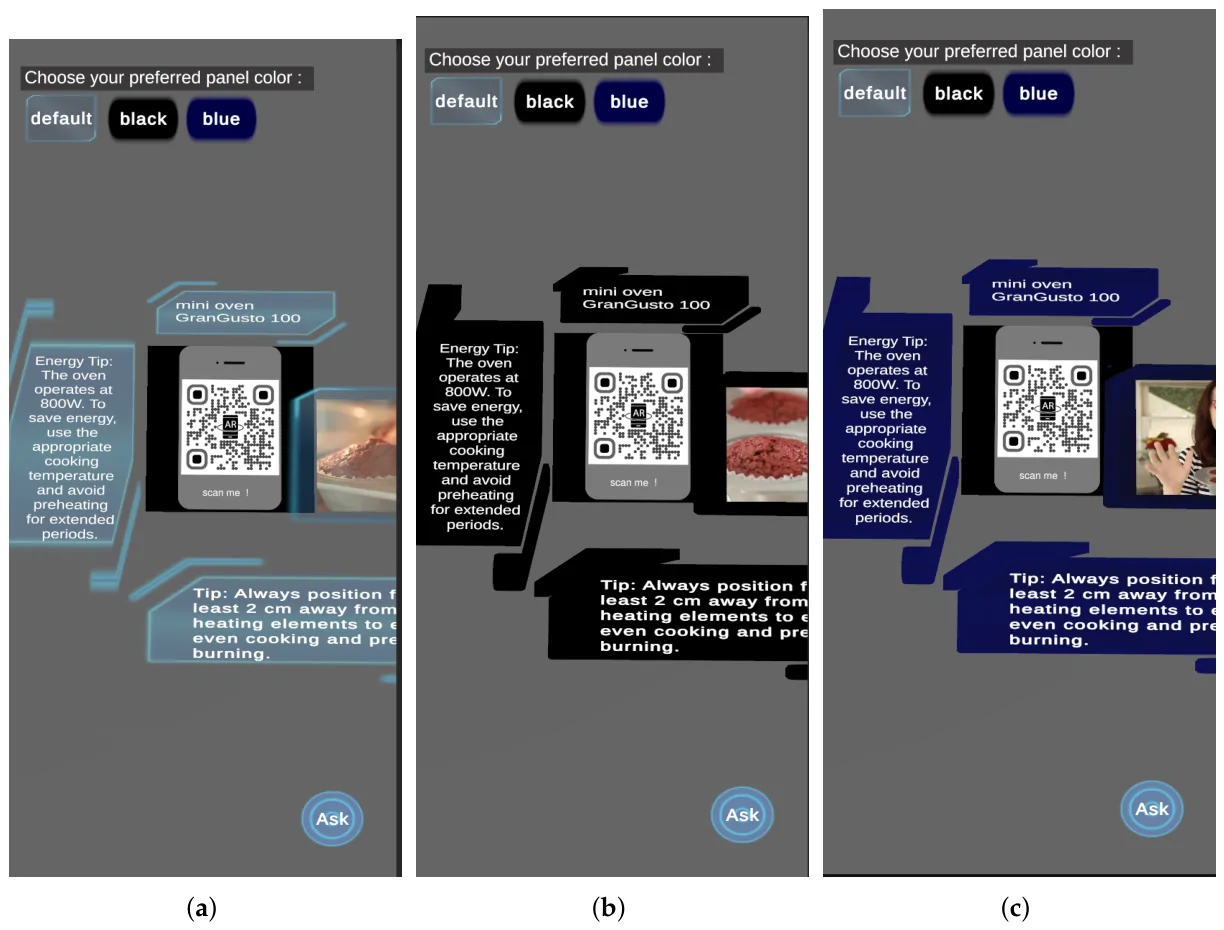
Customizing colors for AR panels to increase readability: (**a**) Default color for AR panels. (**b**) Black color for AR panels. (**c**) Dark blue color for AR panels.

**Figure 15:**
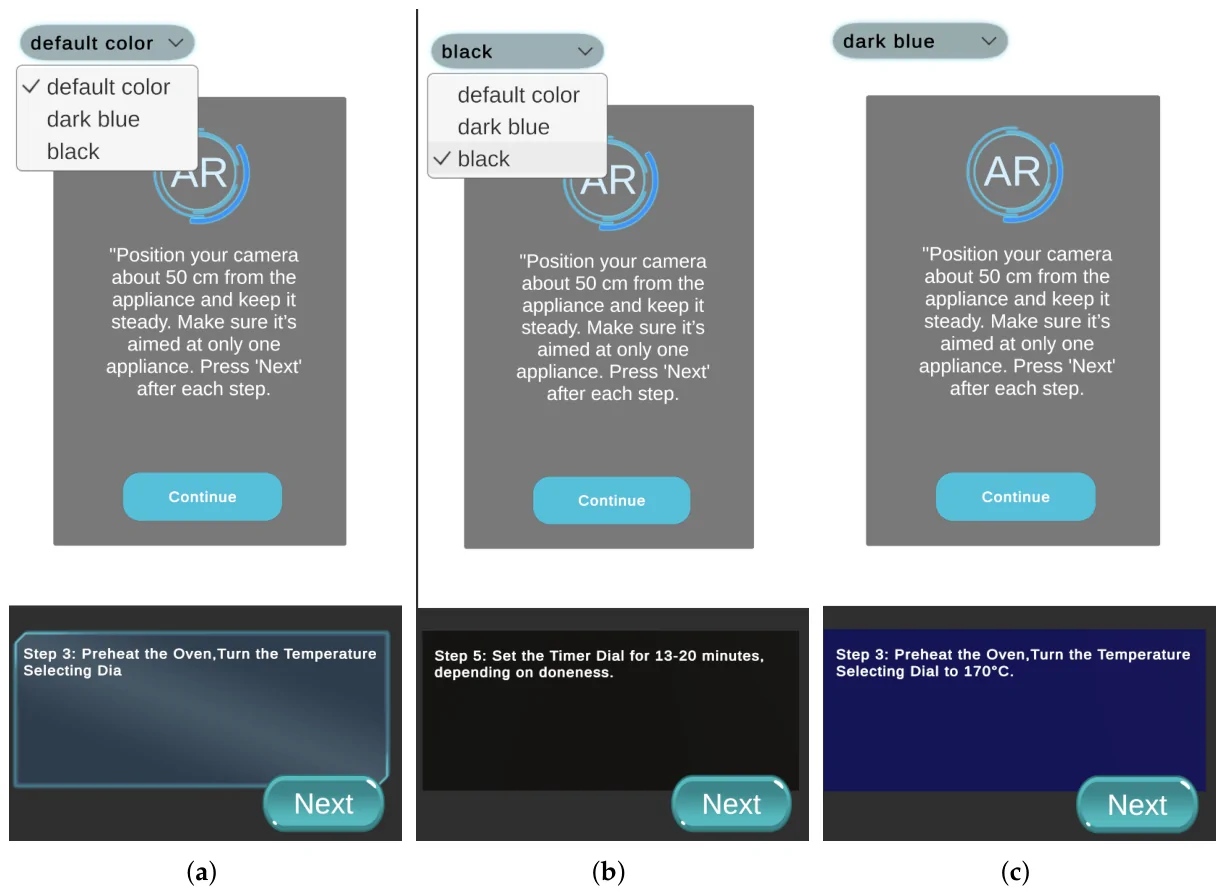
Customizing instruction text background colors to improve readability through a drop-down menu: (**a**) Default color. (**b**) Black color. (**c**) Dark blue color.

The appliance’s color and appearance are carefully crafted to consider different aspects of a diverse range of users. For backgrounds, Neutral gray tones were used to minimize glare and visual distraction. For step-by-step guidance during real-time detection, button detections and dials are highlighted with vibrant colors (e.g., yellow or orange) to direct attention. These colors were selected based on making them easily recognizable. For users with cognitive impairments, to reduce the cognitive load, the text and instructions are as short as possible. Instructions are presented step by step, avoiding complex terminology or multitasking requirements. Furthermore, AR overlays and instructions remain visible until the user completes the current step. To reduce the chance of users forgetting the task they are performing. While prioritizing accessibility, the application is also designed based on a clean, modern appearance to be accepted by the general population. Smooth animations and polished visuals provided a professional look. The color selection feature was included as an accessibility-oriented design option, particularly for improving text readability in different visual conditions. Inside specific scenes of the application, users can choose between three text background colors in AR panels and instruction fields to increase the text readability based on their preference. The three provided options are dark blue(#000066), pure black(#000000), and the default color theme, which is designed for normal users. The text color is always white, and the background colors are chosen based on research done on visual accessibility for people with color blindness and low vision. The World Wide Web Consortium (W3C), in the Web Content Accessibility Guidelines (WCAG 2.1) specifies that a minimum contrast ratio of 4.5:1 is required for text to be readable against a background [51]. Pure black provides a maximum contrast ratio of 21:1, which is more than sufficient. Therefore, it is widely used for high-contrast interfaces, and it is suitable for users with low vision [52]. Using gray or lighter colors instead of black can impact the visibility of text for low-visibility users with low contrast sensitivity. On the other hand, a pure black background can cause increasing strain for some users because the white text appears too harsh. Since blue shades provide softer contrast, dark blue is provided as an alternative for a black background while it offers high contrast with white text and, at the same time, reduces eye strain. Particularly for users with photophobia or contrast sensitivity issues, dark blue is a better choice in terms of contrast differentiation. Additionally, from the color blindness point of view, the dark blue color remains distinguishable andmay improve distinguishability for some users with color-vision differences [53]. Among all types of color blindness, including protanopia, deuteranopia, and tritanopia, red-green and blue-yellow pairings are problematic. Therefore, the pair of a dark blue background and white text, and a pure black background and white text are universal choices. The color selection part of the application in different scenes is shown in Figure 14 and Figure 15.

## 4. Results and Discussion

This chapter contains the technical evaluation of the trained YOLOv8 model for object detection and segmentation, object tracking, the qualitative evaluation of the Gemini results for button descriptions and instruction generation, and the practical usability evaluation of the augmented reality (AR) interface. The validation of the YOLO object detection model is done with quantitative measurements. The trained YOLOv8 models are evaluated based on standard object detection metrics, including mean average precision (mAP@50 and mAP@75), precision, recall, Intersection over Union (IoU), F1-score, and inference time. With these metrics, the model’s ability to classify the buttons and localize them is assessed. In addition to the numerical metrics, the main error patterns and failure cases are discussed because missed buttons or false detections can affect the reliability of the AR guidance. The assessment of the Gemini API is done based on qualitative observations. Evaluation of Gemini in this chapter is qualitative, based on the button description, generated instruction, and how well these data can be visualized in the image for the user. Furthermore, the usability and effectiveness of the developed AR-based appliance guidance system are evaluated based on the designed survey. In the following subsections, the evaluation is provided with details, performance indicators, related charts and tables, and corresponding analyses in the experiments regarding the primary goal of the research. Additionally, the necessary definitions are provided in each section.

### 4.1. Technical Evaluation of Deep Learning Models

This section is dedicated to evaluating the performance of the two trained YOLO models, YOLOv8n and YOLOv8s. As mentioned earlier, in the first step, YOLOv8s was trained on the dataset created by the authors of [13], and this model became the base model for further training. After having this model, the YOLOv8n and YOLOv8s were fine-tuned on the custom dataset created in this work. In addition, the YOLOSeg model was fine-tuned on the proposed custom dataset. The evaluation and results of these three YOLO models are presented here.

#### 4.1.1. Evaluation of YOLOv8 Nano

The YOLOv8n was fine-tuned on 120 epochs on the custom dataset, made by us as discussed earlier. Based on Table 7, the YOLOv8n is a lightweight model, which makes it suitable for use in applications where resources are limited. Also, the low inference time of 0.8 ms per image shows that the model is computationally efficient for the goal of this work, which is using this model in real-time applications, and makes it an acceptable model for the aim of this work.

The result of the model, evaluated on the test set, is shown in Table 8. In this evaluation, precision indicates how many predicted controls are correct, recall indicates how many real controls are detected, F1-score summarizes the balance between precision and recall, and mAP@50 measures the mean average precision when the predicted and true bounding boxes overlap by at least 50%.

**Figure 16:**
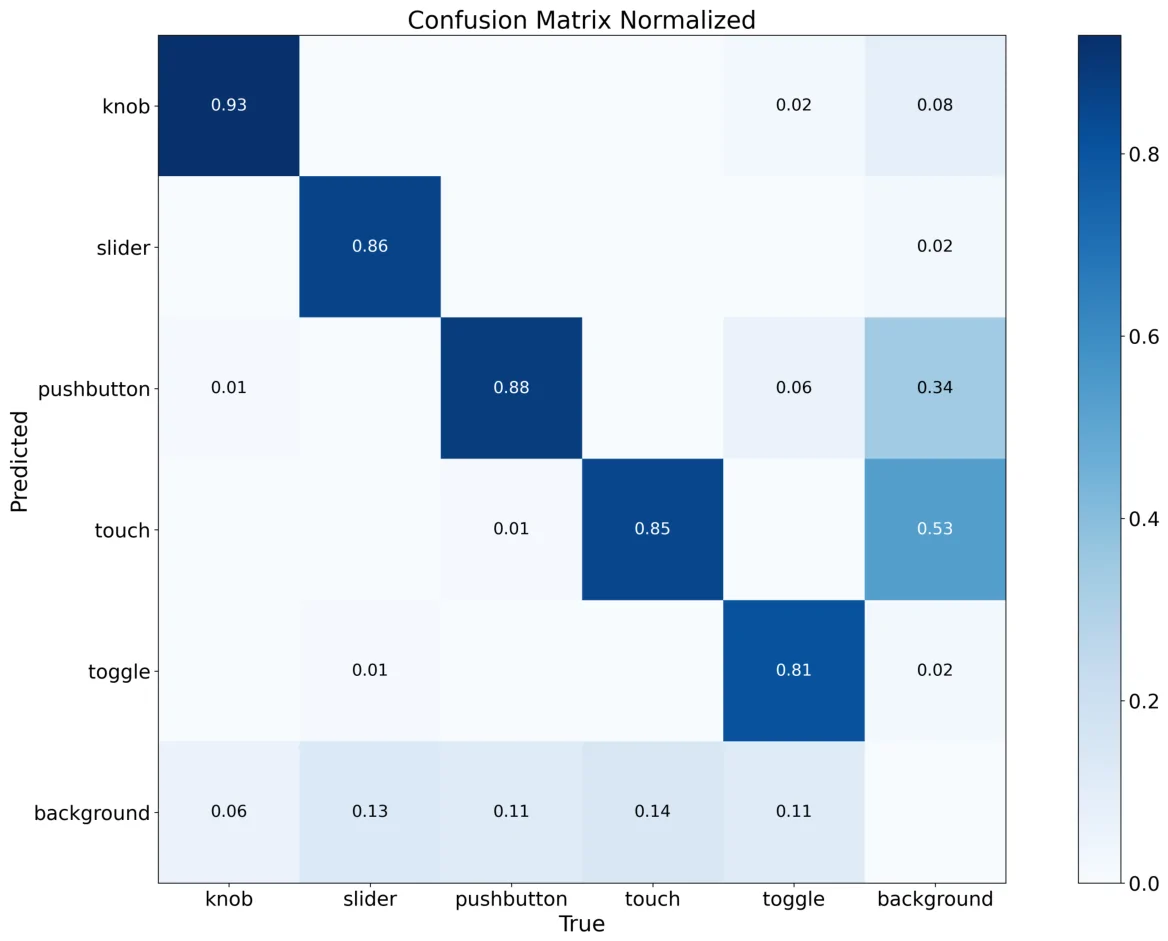
YOLOv8n Normalized Confusion Matrix.

The row that is dedicated to the overall performance of the model shows a high precision of 92.6%, which means that the model generates a low number of false positives; this indicates that most predicted button detections are likely to be correct. However, the value of 84.7% for the recall is lower than the precision, and it results in a model that has slight difficulty in finding some buttons. Overall, the model mAP@50, which is 91.3%, shows acceptable performance for the technical scope of this prototype, indicating that the model is accurate enough to detect and localize the buttons when the IOU threshold is considered 0.5. When we evaluate each class separately, the push button and knob classes have the mAP score of 92.9% and 95.2%, which means that these two classes can be easily detected by the trained model. This is due to the fact that push buttons and knobs are two kinds of buttons that have very clear structures and features, and so the model can see them easily. On the other hand, the drop in the mAP of the classes, like the touch button, indicates that these buttons are hard to detect while within acceptable values for the aim of this work. Also, the normalized confusion matrix of this model is shown in Figure 16.

Analyzing the confusion matrix shows how the model performs at distinguishing the different classes of buttons. The class with the highest accuracy is a knob, with a value of 93%, meaning that the model has a strong ability to recognize the knobs. The slider, push button, and touch button, with 86%, 88%, and 85%, show some difficulties in detection. Also, the toggle is classified correctly 81% of the time, which is the lowest score among all the other classes, but it is still acceptable. The drop in the accuracy of the slider, and specifically the toggle, can come from the fact that they are the underrepresented classes in this work, and the number of samples for these classes in the dataset is lower than that of the other classes. About the misclassification, it seems that the touch buttons are 53% mixed into the background, and push buttons, 34% of the time, are classified as background. This means that it is challenging for the model to detect the touch areas since they sometimes fade into the background. Except for the touch button, which shows some challenges in the missclassification as a background, in other cases, the situation is stable. So it can be concluded that the model performed better in finding the knobs, sliders, and push buttons, and it has some challenges in finding the touch buttons, which are expected due to the nature of these buttons.

Overall, the confusion matrix of this model indicates that the YOLOv8n performs well, and just some modifications in training and a number of images should be made for improvement. For further improvement, adding the extra images to the dataset for the class with lower instances will definitely improve their accuracy, and for the touch buttons, using some augmentation techniques that can highlight these buttons and highlight them for the model can be helpful.

#### 4.1.2. Evaluation of YOLOv8 Small

In this work, the YOLOv8s was fine-tuned for 120 epochs on the custom dataset, which was created. This model has 11,127,519 parameters and is significantly bigger than YOLOv8n, but the hope was that by fine-tuning this model, better results would be achieved. The number of parameters of the YOLOv8s, along with its computational complexity, is shown in Table 9.

The low inference time of the model, along with its high accuracy, makes it suitable for tasks where the response time is crucial, like the systems based on AR proposed in this work. The low inference time of the model, together with its detection accuracy, makes it suitable for further integration in AR-based guidance applications where response time is important. However, the inference time reported here refers to the model-level processing time under the experimental hardware conditions and does not include the full end-to-end latency of the mobile application, camera acquisition, data transfer, Gemini processing, or Unity rendering.

The performance of the model on the test dataset is shown in the Table 10. The same evaluation metrics defined in the previous subsection are used to evaluate YOLOv8s.

The model has an overall precision of 93.4%, showing a good ability to detect the buttons with a relatively low number of false positive detections. However, the recall is 85.7%, and this indicates that some buttons are still missed, especially in classes such as slider and toggle. The overall mAP@50 of the model is 91.4%, which shows acceptable localization and classification performance for the technical scope of this prototype. When the classes are evaluated separately, the knob class achieved the highest mAP@50 of 0.966, which is related to the clearer visual structure of knobs. Lower values for slider and toggle can be related to the lower number of samples and the higher visual diversity of these controls. The rest of the classes, with precision values around 0.93, have acceptable results.

**Figure 17:**
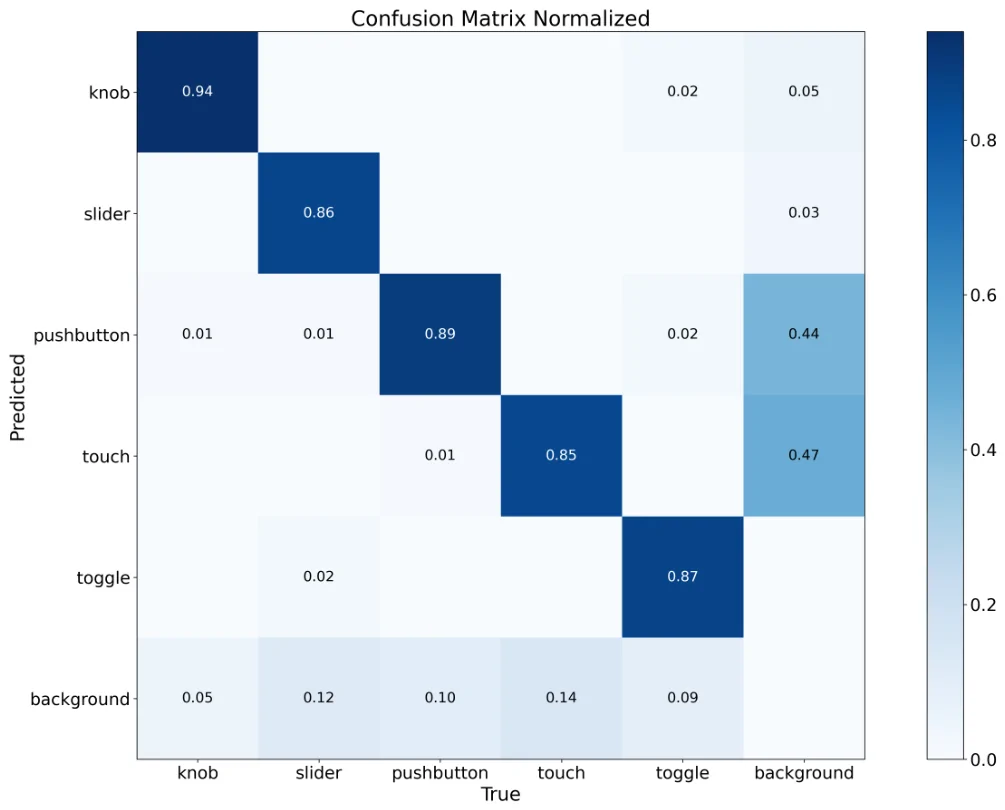
YOLOv8s Normalized Confusion Matrix.

For more in-depth analysis, the normalized confusion matrix of the trained model is shown in Figure 17. Based on the diagonal numbers in the confusion matrix, this trained model has a significant performance in detecting the knobs with an accuracy of 94%, and push buttons have an accuracy of 87%, which, although it is lower than the knob, is a strong performance. The decrease in the value of the toggle and slider classes can be based on their lower number of images in the dataset, and also, sliders are very diverse in their shapes in the applications. The touch button has the lowest accuracy of 85%, meaning the model has a challenge in distinguishing them. About the misclassification, a high number of push buttons and touch buttons are classified as background; this is where the model cannot distinguish them. This misclassification of these types of buttons can be because of their small sizes in the image, and also, in the images of the appliance, the touch and push buttons are very near to each other, and sometimes are not visible to the model to distinguish them from the background. For the rest of the classes, there is no visible issue with misclassifications.

With all the above evaluations, YOLOv8s is a proper model for use in AR guidance applications since it has a low inference time of 1.2ms per image, a low number of misclassifications, and on top of that, the high mAP@50 and robustness of the model.

**Comparative Discussion and Analysis: YOLOv8n and YOLOv8s**

In this part, the YOLOv8n (nano) and YOLOv8s (small) models are compared to each other based on their performance metrics, inference time, and suitability for the task of detecting buttons on the appliances. YOLOv8 shows that it is effective in terms of speed and resource efficiency, while YOLOv8s has superior precision and recall. A comparison of key metrics between YOLOv8n and YOLOv8s is summarized in Table 11 as follows:

This table highlights the difference between these two models based on the different performance metrics. Although YOLOv8n has a faster inference time and needs lower computational resources, YOLOv8s shows better accuracy, precision, and recall, and is more suitable for the goal of this project, which is having high detection accuracy. In total, YOLOv8s has higher precision and recall, enabling it to detect the true positives while minimizing the false positives. This is a critical point and shows its difference, especially in classes like touch buttons, whose detection is challenging for the model. With all the evaluations in hand, the YOLOv8s is the most suitable model for this work because of its high accuracy and class-wise performance, even if it needs more computational cost. Especially in the case of working with people with impairment, the model should be robust, and achieving high accuracy and lower mistakes is more valuable than the computational costs.

#### 4.1.3. Failure Case Analysis and Deployment-Oriented Interpretation

Although the quantitative metrics show that the YOLOv8s model achieved acceptable detection performance for the scope of this prototype, the confusion matrices also show that some failure cases remain important for AR-based guidance. The most relevant errors are related to missed detections, especially when buttons are small, low-contrast, partially occluded, or visually similar to the appliance background. Touch buttons are particularly challenging because they often have weak physical borders and can be printed directly on glossy or dark panels. In such cases, the model may classify them as background or may detect them with lower confidence.

These errors are important because, in a guidance system, a missed button can prevent the application from highlighting the correct control, while a false positive can confuse the user or lead to an incorrect visual indication. For this reason, the model output should not be used as an isolated decision in safety-relevant interactions. In the current prototype, the detection results are combined with the step-by-step instruction pipeline, and future versions should include a confidence-based fallback. If the confidence score is below a predefined threshold, the system should ask the user to adjust the camera position, improve lighting, or confirm the relevant control before continuing the instruction.

The failure cases observed in this work were mainly associated with reflections on glossy surfaces, low contrast between the control and the background, small touch buttons, and dense control panels where push and touch buttons are close to each other. These results suggest that future testing should report class-specific false positives and false negatives under real mobile deployment conditions and should evaluate the system on a held-out set of appliances, lighting conditions, and domestic environments not represented during training.

#### 4.1.4. Evaluation of YOLO-Seg

The evaluation of segmentation includes the analysis of mask precision (P), recall (R), and mean average precision at IoU 0.5 (mAP50). Mask Precision evaluates the proportion of correct segmentation pixels with regard to all predicted masks. Mask mAP measures the overlap between the predicted segmentation mask and the ground truth mask using Intersection over Union (IoU).

The results of model performance are shown in Table 12, and they demonstrate reliable accuracy. The table shows that the trained YOLO-Seg model with Mask mAP50: 90.3% has a high segmentation accuracy at an IoU threshold of 0.5. The overall precision was 0.901, indicating a low false positive rate, and the recall was 0.853, confirming the strong ability of the model to detect instances.

Based on the table above, the overall segmentation performance of the model shows acceptable accuracy for the scope of this work. The mask mAP@50, which measures how well the predicted segmentation masks match the ground truth annotations at an IoU threshold of 0.5, is 90.3% in this model. This value indicates good segmentation accuracy. Also, the mask precision of 0.901 means that there is a relatively low number of false positive segmentation predictions. Finally, the recall value of 0.853 shows the model’s ability to detect relevant instances. The corresponding overall mask F1-score, calculated from precision and recall, is approximately 0.876. In the class-wise evaluation, the knob has the highest mask mAP@50, which is 96.5%, while the toggle class has the lowest mask mAP@50 and the touch class has the lowest recall. This indicates that touch buttons are more challenging to segment because of their similarity with the background and the weak visibility of their edges on appliance panels.

**Figure 18:**
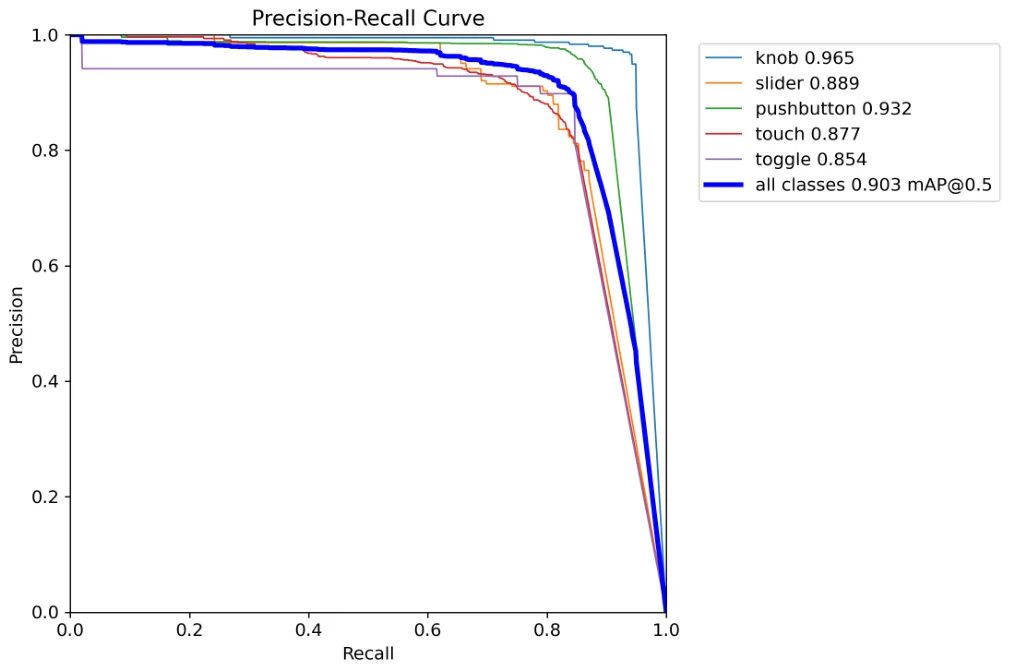
YOLOv8-seg PR Curve.

Precision-recall curve, which is demonstrated in Figure 18, shows the trade-off between precision and recall across different confidence thresholds. The overall precision of the model is high, especially for the knob and push button class. The most challenging part is the touch buttons, where their precision drops when recall increases. Overall, the precision-recall value among all the classes is acceptable for the scope of this work.

**Figure 19:**
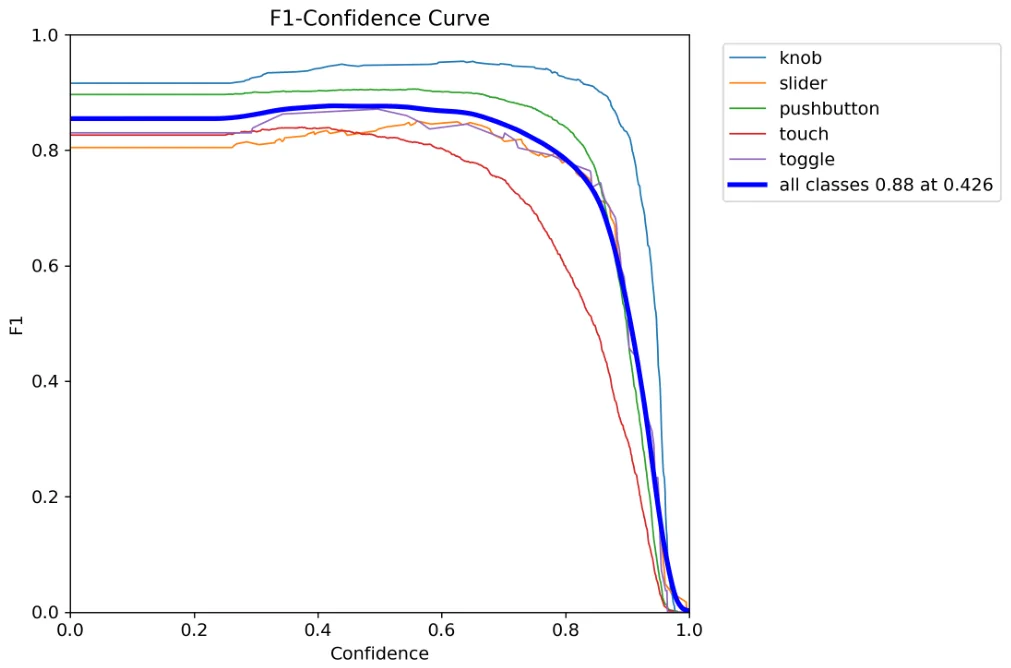
YOLOv8-seg F1 Curve.

The F1-confidence curve of this model is shown in Figure 19. From this curve, it can be obtained that the highest F1 score of 0.88 can be achieved at the confidence level of 0.426. However, the touch button and sliders have declined in their F1-score at the higher confidence thresholds, due to the fact that more misclassification is occurring for these two classes at higher confidence thresholds.

### 4.2. Gemini Evaluation

Two important tasks of Gemini in this work are describing the buttons based on their attributes and generating a step-by-step instruction for the user. In this section, the responses of Gemini in these two areas are evaluated. Since, for this part, there is no ground truth for giving the quantitative results, the Gemini performance will be evaluated in a qualitative manner by manual verification.This qualitative evaluation was focused on manual consistency, completeness of the generated steps, relevance to the detected or described button, and the absence of clearly unsupported or unsafe instructions. Therefore, the results should be interpreted as a preliminary verification of the language-model component, rather than as a full benchmark of response accuracy or safety. Because the language model is responsible for transforming manual information into step-by-step guidance, its output was checked with particular attention to manual grounding and safety. In the present proof-of-concept version, the verification was performed qualitatively by reviewing whether the generated responses were consistent with the uploaded user manual, whether the main steps were complete, whether the answer avoided unsupported appliance functions, and whether the suggested action could be connected to the detected or described control. This verification was mainly used to identify obvious hallucinations, missing steps, unsafe suggestions, and incorrect button mapping.This evaluation should be considered preliminary because no independent benchmark of correct manual-grounded answers was available for all tested appliances. For this reason, the system was designed to use conservative behavior when uncertainty occurs. If the manual does not provide enough information, or if the control cannot be mapped with sufficient confidence, the system should avoid giving a final operational instruction and should ask for user confirmation or a clearer camera view. A more systematic evaluation with expert-coded accuracy, completeness, safety, manual grounding, and button-mapping correctness is required in future work.

**Buttons attribute description** As mentioned in previous sections, Gemini was asked to determine the attributes of the button, including shape, relative position, appearance, and functionality, based on the user manual. These attributes will help the user to operate the appliances more easily, especially in cases where impaired users need additional guidance. Gemini, in this part, will get the cropped button along with the user manual and generate the descriptions. The responses generated by Gemini are evaluated based on the user reviews to ensure the accuracy and reliability of the responses. The following points should be considered when the responses are analyzed:

- **Completeness of the Response**: The evaluation verifies that all relevant attributes—such as shape, color, appearance, and button name—are explicitly included in the output generated by the Gemini model for each detected button.
- **Consistency**: It is essential that the descriptions follow the predefined format specified in the input prompt to Gemini, ensuring structural uniformity across all button outputs.
- **Relevance**: Each button’s description must accurately pertain to the corresponding button instance, without including attributes or details that belong to other detected buttons.

**Figure 20:**
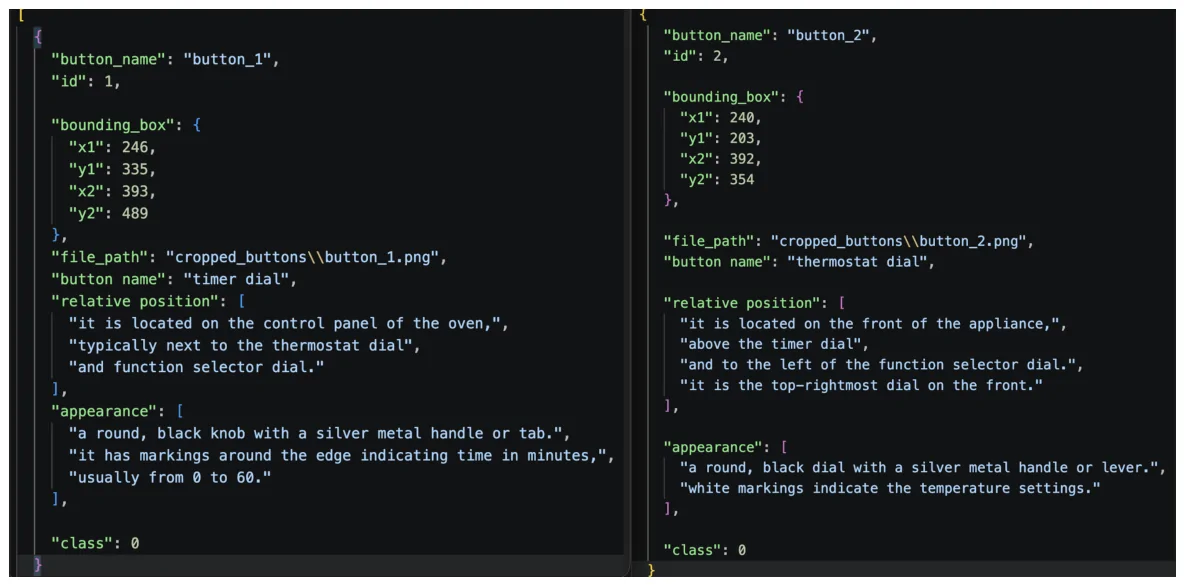
Description of buttons generated by Gemini.

As shown in the Figure 20, Gemini was able to provide the button attributes in a short time, with no major missing information observed in the evaluated cases.However, because this assessment was based on manual review and not on an independent annotated ground truth, the reliability of button descriptions and instruction generation should be further evaluated in future work using expert ratings and inter-rater agreement.

**Instruction generation** Another task that is done by Gemini is to get the user manual as a PDF file, and also get the user query, and based on these two answers to the user’s query. Gemini is asked to generate step-by-step instructions to guide the user through their need. The result is expected to be an instruction in which, in each step, the name of the button and the required action are specified. Also, the structure of the response is like what is defined for the Gemini in the sent prompt. For evaluating the instruction, the following points should be checked:

- **Accuracy**: The generated instruction must align with the actual functionality of the appliance as documented in its official user manual and reflect realistic operational behavior.
- **Clarity**: The instruction should be expressed in a clear, concise, and unambiguous manner, ensuring it is easily understood by the user.
- **Completeness**: All essential procedural steps should be present in the instruction to avoid incomplete guidance or omissions that may hinder successful task execution.

**Figure 21:**
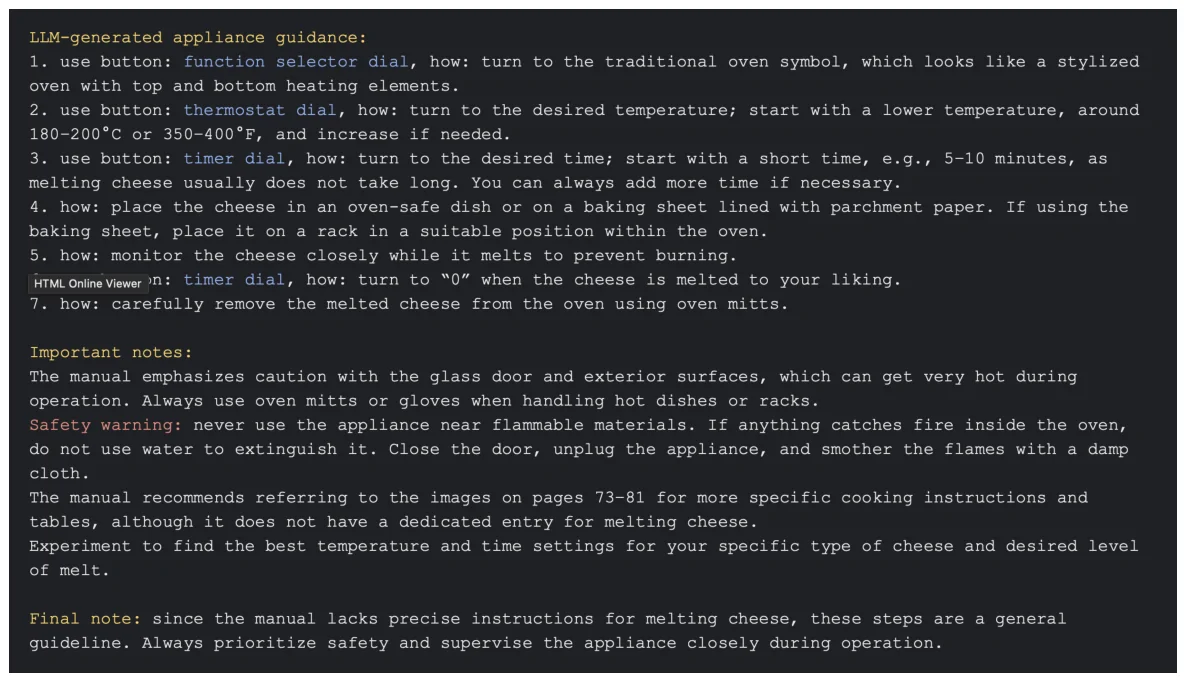
Example of the final structured instruction generated by Gemini for the selected user request.

In the Figure 21, the sample of the generated instruction by Gemini is demonstrated, and it proves that Gemini can effectively give instructions in a detailed and structured way.

### 4.3. AR Application Evaluation

The AR application was evaluated through a preliminary task-based comparison and a post-use questionnaire. A total of 20 participants completed the evaluation. The participants were recruited from the general population and most of them were between 27 and 45 years old, while a smaller number of older participants were also included. Two participants self-reported color blindness. Digital literacy and prior experience with AR applications were self-reported. No formal cognitive screening, visual acuity assessment, disability assessment, or clinical diagnosis verification was conducted. Therefore, the evaluation should be interpreted as a non-clinical usability and proof-of-concept assessment. Table 13 summarizes the demographic characteristics of the study participants.

For the task-based comparison, participants were asked to complete the same oven-related task under two conditions: a manual plus recipe-search condition and the proposed AR application condition. The selected task required the user to identify how to melt a specific type of cheese using the tested oven model. Since the appliance manual provides oven-specific information about functions, buttons, and settings, but does not necessarily provide recipe-specific information such as the appropriate temperature and time for melting a particular cheese, participants in the manual condition were allowed to consult both the paper manual of the exact oven model and an external recipe source, such as an internet search or a recipe book. In the application condition, participants used the proposed system, starting from QR-code scanning, followed by manual retrieval, language-model-based instruction generation, and step-by-step AR guidance.

Task completion time was measured from the moment the participant started searching for information until the final appliance setting was identified and applied. In the manual plus recipe-search condition, timing started when the participant received the task and began consulting the paper manual or external recipe source. In the application condition, timing started when the participant began QR-code scanning and ended when the system provided the step-by-step instruction and the participant identified the required oven control and setting. Observed task errors, task success, and perceived workload were also recorded.

Table 14 reports the exploratory task-based comparison between the manual plus recipe-search condition and the AR application condition. Because the study was not powered as a confirmatory experiment, these results are interpreted descriptively. The table emphasizes the magnitude and practical direction of the observed differences rather than formal evidence of efficacy. Where participant-level paired data are available, future versions of this evaluation should report the paired mean difference, 95% confidence interval, and standardized within-participant effect size for each main outcome.

The observed changes are descriptive and should be interpreted as preliminary task-based feasibility indicators. The present table does not provide confirmatory evidence of efficacy. Fully paired confidence intervals and standardized within-participant effect sizes require participant-level paired data for each outcome.

The perceived workload score was a study-specific single-item rating on a 0–100 scale and should not be interpreted as a validated multidimensional cognitive-workload measure. In particular, cognitive workload was not assessed with a validated instrument such as NASA-TLX.

The task-based results showed that the AR application condition was associated with shorter completion time, fewer observed errors, lower perceived workload, and higher task success than the manual plus recipe-search condition. The mean completion time decreased from 10.8 min in the manual plus recipe-search condition to 4.2 min in the AR application condition. This application timing is consistent with the recorded prototype videos, in which the process from QR-code scanning to receiving step-by-step guidance generally required approximately three to five minutes.

Although this comparison provides a more task-oriented evaluation than the questionnaire alone, the results should still be interpreted cautiously. The sample was small and non-clinical, and no formal cognitive screening, visual acuity assessment, disability-status assessment, or clinical diagnosis verification was conducted. Therefore, the findings should be interpreted as preliminary evidence of task performance and usability in a proof-of-concept setting, rather than evidence of clinical effectiveness.

After completing the application-based task, participants completed a questionnaire divided into five key areas. This questionnaire was used to assess each major interaction stage separately and to identify possible areas for improvement. Here are the five key areas of the survey that will be discussed in detail later.

- **Welcome and Introduction:** Assessment of the initial user experience, including clarity of instructions and interface engagement.
- **QR Code Scanning Process:** Evaluation of the ease and reliability of scanning the appliance-specific QR code.
- **Appliance Marker Detection and Information Display:** Measurement of system responsiveness and accuracy in identifying the appliance and presenting relevant information.
- **User Query Answering and Visualization:** Examination of how effectively the system responds to user queries and provides appropriate visual guidance.
- **Overall Usability and User Satisfaction:** General evaluation of the application’s user-friendliness, accessibility, and perceived usefulness.

Each section contained questions where participants rated their experience on a scale from 1 (Strongly Disagree) to 5 (Strongly Agree). The following statements were presented to participants for rating:

**Welcome and Instructions of Application**

- The welcome screen offered clear and concise guidance for initiating the application.
- The instructional messages were comprehensible and easy to follow, facilitating smooth user onboarding.
- The positioning and functionality of the “Continue” buttons were intuitive and easily accessible, contributing to seamless navigation.

**QR Code Scanning**

- The QR code scanning process was intuitive and required minimal user effort.
- The on-screen instructions provided for scanning the QR code were clear and effectively guided the user.
- The system accurately and promptly detected the appliance’s QR code using the device camera, ensuring a seamless transition to the next interaction stage.

**Appliance Marker Detection and Information Display**

- The appliance marker detection was performed with high speed and accuracy, facilitating an efficient AR experience.
- The augmented reality overlays—comprising appliance-specific tips, instructional videos, and usage suggestions—were perceived as helpful and informative.
- The content presented through the overlays was relevant to the identified appliance and clearly understandable by users.

**Answering Questions and visualization guidance**

- The Ask button was easily identifiable and intuitive to operate within the user interface.
- The system successfully provided relevant and helpful responses to appliance-related queries submitted by users.
- The answers delivered by the system effectively indicated the specific button to be pressed, enhancing the clarity of instructions.

**Overall Usability**

- The application’s interface was intuitive and facilitated seamless navigation throughout the user experience.
- The augmented reality (AR) features enhanced my comprehension of appliance usage procedures.
- The application performed reliably without noticeable latency or unexpected crashes.
- Overall, I was satisfied with the functionality and usability of the application and would recommend its use to others.

Additionally, participants were encouraged to provide qualitative feedback and suggestions for improvement.No missing questionnaire responses were present; therefore, all 20 participants were included in the descriptive analysis for each item. In the following, the survey results will be presented and analyzed. This includes statistical summaries, such as mean scores and standard deviations, and visualizations of participant responses in the form of bar charts.

No a priori power analysis was conducted because the user evaluation was designed as an exploratory proof of concept usability assessment rather than as a confirmatory clinical, cognitive, or behavioral efficacy study. The sample size of 20 participants should therefore be interpreted as sufficient only for preliminary descriptive and task-based feasibility evidence. As a sensitivity indication, for a paired within-participant comparison with (n=20), (α=0.05), and 80% power, the minimum detectable standardized effect is approximately ($d_{z}=0.66$). Therefore, the present evaluation was suitable for detecting only medium-to-large within-participant effects and was not powered to detect small effects or to support population-level conclusions. Future confirmatory studies should define a primary outcome in advance, such as task-completion time, observed error rate, SUS, NASA-TLX, or confidence ratings, and should calculate the required sample size accordingly.

For this reason, the user-evaluation results were interpreted primarily in terms of descriptive magnitude, practical relevance, and statistical uncertainty. For the task-based comparison between the manual plus recipe-search condition and the AR application condition, completion time, observed errors, perceived workload, and task success were summarized for both conditions. Where participant-level paired data were available, within-participant differences were reported together with 95% confidence intervals and standardized effect-size estimates. For approximately normally distributed paired differences, Cohen’s ($d_{z}$) was used, calculated as the mean paired difference divided by the standard deviation of the paired differences. For ordinal or non-normally distributed outcomes, descriptive paired differences and non-parametric effect-size measures were preferred. Task success was reported as the number and percentage of participants completing the task in each condition.

Questionnaire responses were analyzed descriptively at item level and scene level. For each questionnaire item and interaction scene, mean, standard deviation, and 95% confidence interval were reported when the available participant-level data allowed this calculation. Because the questionnaire was study-specific and was not a validated psychometric instrument, these results were interpreted as preliminary subjective usability feedback, not as evidence of validated cognitive workload reduction, clinical efficacy, or accessibility benefit in target populations.

**Statistical Analysis and Visual Results**

Questionnaire responses were analyzed descriptively using mean Likert ratings and standard deviations. The results are shown in Table 15. The mean scores, calculated from participant responses on a scale from 1 to 5, reflect subjective ratings of clarity, ease of use, responsiveness, visual guidance, and overall satisfaction. The standard deviation (SD) was computed to describe the variability of participant responses for each interaction scene.

**Figure 22:**
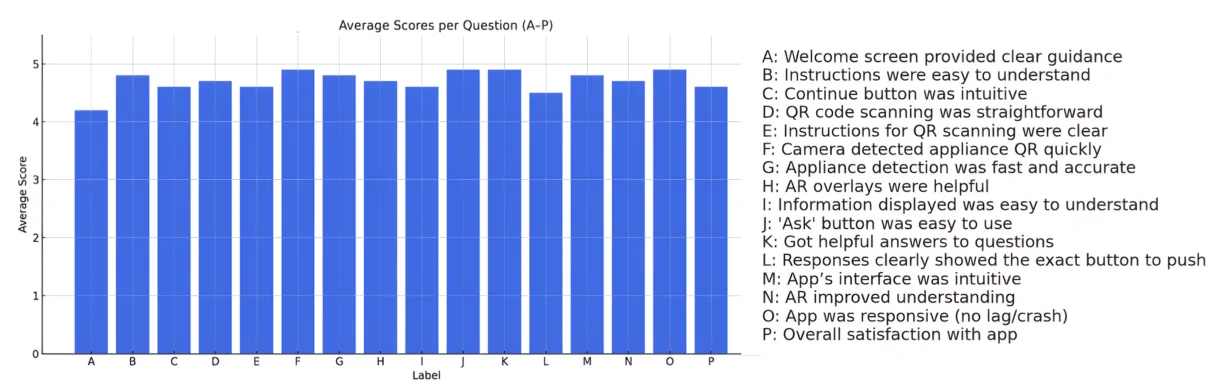
Mean Likert ratings across all questionnaire items. The labels A–P correspond to the questionnaire items listed in the figure legend.

The bar chart in Figure 22 illustrates the mean Likert ratings across all questionnaire items. The labels A–P correspond to the individual questionnaire statements presented to participants.

The questionnaire results showed generally positive subjective feedback across all application stages. The highest scene-level score was observed for the question-answering and visual guidance stage, with a mean score of 4.77. This suggests that participants found the system responses and button-level visual guidance useful for completing the appliance task. Overall usability, QR code scanning, and appliance detection with AR overlays also received high mean scores of 4.75, 4.73, and 4.70, respectively. The lowest scene-level score was observed for the Welcome and Introduction stage, although it remained positive with a mean score of 4.53. This suggests that the initial onboarding instructions could still be simplified, especially for participants with lower digital literacy. The mean SD values were below or close to 0.5 across the interaction scenes, suggesting relatively consistent responses among participants.

### 4.4. Theoretical Interpretation for Cognitive Accessibility

From a psychological and neurocognitive perspective, the proposed system may be interpretednot merely as a visual assistance tool, but as a possible external cognitive scaffold for instrumental daily activities. Operating modern appliances requires the coordination of several functions that are frequently vulnerable in ageing and mild cognitive impairment, including selective attention, visuospatial search, working memory, action sequencing, and error monitoring. Traditional manuals place a substantial burden on these processes because users must continuously translate abstract written instructions into situated actions on a physical interface. By contrast, the present AR-based approach is designed to externalize part of this cognitive work: it narrows the action space, visually emphasizes the relevant control, and decomposes procedures into manageable sequential steps. In theoretical terms, this may reduce extraneous cognitive load, lower uncertainty during action selection, and increase perceived self-efficacy. These aspects are especially relevant for older adults and users with early cognitive vulnerabilities, for whom difficulties in instrumental activities of daily living often emerge not only from memory impairment per se, but also from the interaction between reduced executive control and complex technological environments. Recent developments in Ambient Assisted Living systems have similarly emphasized the importance of adaptive artificial intelligence and context-aware human—computer interaction to support vulnerable users through personalized multimodal assistance and cognitively supportive environments [54]. At the same time, the present findings should be interpreted cautiously. The task-based comparison with the manual plus recipe-search condition provides preliminary support for improved task performance in the tested scenario, as participants completed the task faster, made fewer observed errors, and reported lower perceived workload when using the application. However, the evaluation was conducted with a small non-clinical convenience sample, and no formal cognitive screening, visual acuity assessment, disability-status assessment, or clinical diagnosis verification was performed. Therefore, the present results support preliminary usability, acceptability, and task-performance feasibility, but they do not demonstrate clinical efficacy, cognitive benefit, or accessibility effectiveness in populations with diagnosed visual or cognitive impairment. Future studies should directly assess the system with older adults and users with confirmed cognitive or visual limitations, using validated workload and usability instruments, task-completion measures, error analysis, and longer-term retention or transfer tasks.

### 4.5. Discussion of Technical Feasibility, Safety, and Future Validation

The results of this work mainly show the technical feasibility of integrating different components into one AR-based appliance guidance system. The trained YOLOv8 model was used for detecting appliance controls, YOLO-Seg was used for visualizing the detected controls with segmentation masks, and BoT-SORT was used to keep the detected buttons stable during the real-time camera interaction. In addition, the Gemini API was used to extract information from the user manual and generate step-by-step instructions, while the Unity application visualized the guidance on the camera view. Therefore, the main demonstrated contribution of this work is the integration of detection, segmentation, tracking, manual-grounded instruction generation, and AR visualization in a single proof-of-concept pipeline. The preliminary user evaluation also suggested that the interface was understandable and acceptable for the non-clinical participants who tested the prototype.

From the perspective of cognitive accessibility, the system can be considered relevant because it gives instructions directly on the appliance interface and reduces the need to search manually through long user manuals. The visual highlighting of the detected controls and the step-by-step presentation of the instructions may also reduce the need to remember the complete sequence of actions at once. However, these are theoretical mechanisms and were not directly measured in the present study. Cognitive workload, self-efficacy, functional autonomy, and clinical benefit were not evaluated with validated instruments or with a clinical population. For this reason, the present results should be interpreted as technical and preliminary usability findings, while cognitive-accessibility benefits remain hypotheses for future studies.

Another important point is that failure cases are especially relevant in an assistive guidance system. False detections, missed buttons, or wrong matching between an instruction and a detected control may confuse the user and, in some appliances, may lead to unsafe operation. In the current prototype, this risk was partly addressed by grounding the language-model response in the retrieved user manual and by providing the model with the detected button information before generating the step-by-step guidance. The backend instructions also asked the model not to invent appliance-specific settings when the information was not available in the manual. However, touch buttons were among the more challenging controls because they are often small, flat, low-contrast, or visually similar to printed symbols on the panel. In addition, reflections, hand occlusions, low lighting, and camera movement can affect detection and tracking stability. Therefore, although manual-grounding and backend prompting were used as initial safeguards, future versions should include more explicit low-confidence behavior, such as asking the user to adjust the camera position, improve lighting, or confirm the selected control before continuing. The language-model component should also flag uncertainty when a detected control cannot be matched reliably to the manual-based instruction, especially for appliances involving heat, electricity, water, or other safety-sensitive functions.

Before cognitive or clinical claims can be made, a staged validation pathway is required. The first stage is technical validation of detection, segmentation, tracking, and instruction generation. The second stage is non-clinical usability testing with more structured tasks and objective measures such as task-completion time, number of errors, and number of prompts required. The third stage should involve older adults and users with different levels of digital literacy. The fourth stage should evaluate clinical feasibility with people with mild cognitive impairment or other relevant cognitive difficulties. Finally, the system should be compared with standard instructional supports, such as printed manuals, videos, caregiver instruction, or unaided appliance use.

### 4.6. Limitations and Future Work

The present study has some limitations that should be considered when interpreting the results. First, the user evaluation was conducted with a limited non-clinical sample. Therefore, the results can only show preliminary subjective usability, clarity, and acceptability, and they cannot be used to claim clinical benefit or cognitive accessibility in target populations. Older adults, people with visual impairments, people with mild cognitive impairment, and people with Alzheimer’s disease were not directly evaluated in the present study. In addition, cognitive screening, visual-acuity assessment, disability status, and digital-literacy profile were not systematically measured.

Second, the current evaluation does not yet quantify effects on workload reduction, task completion time, error rate, confidence, retention or transfer of learning, or long-term autonomy in real domestic settings. It also does not include a comparator condition with printed manuals, videos, caregiver instruction, or unaided appliance use. Future studies should therefore use structured task-based protocols and include objective measures such as task-completion time, number of errors, number of prompts required, and validated instruments such as NASA-TLX and SUS.

From the technical side, although the detection and segmentation results were promising, deployment in real homes can still be affected by lighting variability, reflections on appliance panels, hand occlusions, cluttered backgrounds, camera movement, and the small or low-contrast appearance of some controls. This is consistent with recent AR research showing that lighting conditions can influence the accuracy and reliability of visual AR systems [55]. Touch buttons may be especially challenging because they are often flat and visually similar to printed symbols. Expanding the dataset and testing the system in more heterogeneous real-world environments will be important for improving robustness and ecological validity.

The use of QR code scanning is another limitation. In this prototype, QR codes were used to retrieve the correct appliance manual and reduce the risk of selecting the wrong model. However, QR scanning may create usability barriers for users with motor, visual, or cognitive difficulties. Future versions should consider complementary entry methods, such as appliance image recognition, barcode or model-number recognition, NFC tags, voice-assisted manual selection, or caregiver-assisted setup.

Although backend prompting and manual-grounding were used to reduce unsupported language-model outputs, the present study did not include a full quantitative evaluation of hallucination, omission, unsafe instruction, or repeated-prompt consistency. Future work should evaluate the language-model component using a curated set of appliance manuals and user queries, with expert-coded ratings of accuracy, completeness, safety, manual consistency, and button-mapping correctness.

Future developments may also include read-aloud assistance, enhanced multimodal feedback, haptic interaction, and adaptive personalization mechanisms based on user behavior and familiarity with the appliance [56]. Moreover, natural and gesture-based interaction paradigms could further improve accessibility and intuitiveness in assistive AR environments by reducing dependence on conventional touch-based interfaces, in line with recent advances in wearable and intuitive human–machine interaction systems [57]. Future studies involving older adults, people with cognitive impairment, or other vulnerable populations should also include appropriate ethics approval, consent procedures, and privacy-preserving data-governance strategies.

## 5. Conclusions

The present study introduced an integrated augmented reality and artificial intelligence-based system for contextual appliance guidance, designed to support users during interaction with complex household and office appliances. The proposed architecture integrates object detection, segmentation, tracking, large language model-based instruction generation, and real-time AR visualization within a unified pipeline. From a technical perspective, the system showed promising performance in button detection and segmentation, while the preliminary user evaluation suggested positive subjective ratings of usability, clarity, and acceptability of the interface in a non-clinical sample.

Beyond its engineering contribution, the proposed system may be relevant from the perspective of cognitive accessibility. By transforming static manual information into situated, visually grounded, and sequential guidance, the system may reduce the need to search through manuals and remember the complete sequence of actions at once. In this sense, the platform can be considered as a possible external cognitive support mechanism, but this interpretation remains theoretical in the present study.

Overall, the findings should be interpreted as a technology-oriented proof of concept rather than evidence of clinical or cognitive effectiveness. Cognitive workload, self-efficacy, functional autonomy, and clinical benefit were not directly measured. Future controlled studies are needed with older adults, people with visual impairments, and people with mild cognitive impairment to evaluate whether the proposed approach can improve real appliance use compared with standard instructional supports. 

## Figures and Tables

**Table 1:** Description of Frame Selection and Clustering Process.

Step	Description
Calculate Sharpness	Computes the sharpness of a frame using the variance of the Laplacian. The frame is converted to grayscale, and edge details are detected. A higher variance value signifies a sharper image.
Calculate Focus on Center	Assumes that the most critical information is located at the center of the frame. The central 50% of the frame’s height and width is extracted, and edge detection (Canny) is applied. The sum of detected edges determines the focus quality.
Calculate Lighting Uniformity	Measures the uniformity of lighting across the frame by computing the standard deviation of pixel intensities in grayscale. A higher standard deviation indicates less uniform lighting.
Frame Scoring and Selection	After computing sharpness, center focus, and lighting uniformity scores, the top 25 best frames are selected based on their cumulative scores.
Cluster Frame Function	Frames are grouped into clusters using edge-based feature extraction. The Canny edge detector extracts edges, which are then converted into 1D vectors and normalized. Finally, K-Means clustering is applied to ensure diverse frame selection.

**Table 2:** Class Distribution Before and After Adding Slider Images.

Class	Before Online Images	After Online Images	Change (%)
Knob (Class 0)	2569	2569	0%
Slider (Class 1)	578	1278	+121%
Push Button (Class 2)	14,392	14,392	0%
Touch Button (Class 3)	14,696	14,696	0%
Toggle (Class 4)	504	504	0%

**Table 3:** Summary of Annotation Statistics.

Source	Total Annotated Images	Total Annotations
Our Captures	2862	18,754
Form Captures	2013	13,985
Amazon & eBay	700	5460
Total	5575	38,199

**Table 4:** Data Augmentation Parameters and Justification.

Parameter	Value	Definition	Justification for Task
hsv_h	0.01	Horizontal perturbation in hue.	Maintains consistent color tones while adding slight variation to handle diverse lighting.
hsv_s	0.6	Perturbation in saturation.	Increases resilience to differences in color intensity, like faded or vibrant appliances.
hsv_v	0.5	Perturbation in value (brightness).	Helps the model generalize across bright and dark image regions.
degrees	3.0	Maximum rotation angle.	Accounts for slight tilts in user-captured images.
translate	0.05	Maximum shift as a percentage of dimensions.	Models positional variability in button placement.
scale	0.3	Scale variation.	Makes the model robust to varying appliance distances or zoom levels.
shear	2.0	Maximum shearing angle.	Simulates slanted camera views.
fliplr	0.5	Probability of horizontal flip.	Reflects mirrored control panel layouts.
mosaic	1	Mosaic augmentation enabled.	Combines multiple images to enrich spatial diversity.
copy_paste	0.2	Object-level copy-paste.	Boosts object count and variability in scenes.
erasing	0.2	Random erasure regions.	Mimics occlusions like hands or reflections.
crop_fraction	0.95	Retained image portion post-crop.	Avoids cutting important panel regions.
auto_augment	“randaugment”	Automatic random augmentation.	Adds diversity without overfitting to manual patterns.

**Table 5:** Qualitative comparison of object tracking methods considered for appliance-control tracking.

Tracker	Type	ReID	Occlusion Handling	Strengths	Weaknesses
Norfair	Centroid-based tracking using object coordinates.	No	Poor	Lightweight and fast for simple scenes.	Cannot re-identify objects once lost due to occlusion.
DeepSORT	Appearance-based tracking using feature embeddings.	Yes	Moderate	Re-identifies objects using deep visual features.	May fail when multiple objects have very similar appearance.
BoT-SORT	Hybrid approach combining ByteTrack, ReID, and global motion compensation.	Yes	Strong	Provides stable tracking and better recovery after temporary occlusion.	Requires more computation and parameter tuning.
FairMOT	Joint object detection and tracking in a unified network.	Yes	Strong	End-to-end multitask learning can improve tracking accuracy.	Computationally expensive for real-time deployment.
ByteTrack	IoU-based matching of high- and low-confidence detections.	No	Limited	Robust when detections are reliable and continuous.	May lose identity during occlusion or camera movement.

**Table 6:** Configuration Parameters Used for Gemini 1.5 Pro.

Parameter	Definition	Effect	Reason for Selection
temperature = 0.2	Controls randomness in output.	Low temperature leads to more deterministic and focused responses.	Ensures factual accuracy, crucial for appliance instruction generation.
top_p = 0.8	Enables nucleus sampling.	Allows a mix of likely and diverse tokens to be selected.	Encourages variety while maintaining correctness.
top_k = 64	Limits token selection to the top-k candidates.	Reduces use of rare or unlikely words.	Balances between creativity and control.
max_output_tokens = 8192	Specifies the maximum length of the generated response.	Allows for complete and lengthy answers.	Required for generating multi-step guides and detailed instructions.

**Table 7:** YOLOv8n Model Parameters and Performance Metrics.

Attribute	Value
Number of Parameters	3,006,623
FLOPs (Floating Point Operations)	8.1 GFLOPs
Inference Speed	0.8 ms per image
Optimizer	AdamW (learning rate = 0.001111, momentum = 0.9)

**Table 8:** Evaluation Metrics for YOLOv8n on Test Dataset.

Class	Images	Instances	Precision (P)	Recall (R)	F1-Score	mAP@50
All	546	3299	0.926	0.847	0.885	0.913
Knob	183	274	0.928	0.923	0.925	0.952
Slider	105	120	0.952	0.825	0.884	0.908
Push Button	312	1587	0.955	0.851	0.900	0.929
Touch	117	1265	0.897	0.787	0.838	0.873
Toggle	42	53	0.900	0.849	0.874	0.902

**Table 9:** YOLOv8s Model Summary.

Attribute	Value
Number of Parameters	11,127,519
Number of Layers	168
FLOPs (GFLOPs)	28.4
Input Image Size	640×640 pixels
Average Inference Time	1.2 ms per image
Postprocessing Time	0.8 ms per image

**Table 10:** Performance Metrics for YOLOv8s on the Test Dataset.

Class	Images	Instances	Precision (P)	Recall (R)	F1-Score	mAP@50
All	1087	7128	0.934	0.857	0.894	0.914
Knob	371	512	0.957	0.936	0.946	0.966
Slider	207	239	0.932	0.808	0.866	0.880
Pushbutton	605	3147	0.931	0.876	0.903	0.929
Touch	273	3122	0.914	0.850	0.881	0.902
Toggle	73	108	0.938	0.815	0.872	0.893

**Table 11:** Performance Comparison Between YOLOv8s and YOLOv8n.

Metric	YOLOv8s	YOLOv8n
Parameters	11,127,519	3,006,623
GFLOPs	28.4	8.1
Inference Time (ms/image)	1.2	0.8
mAP@50 (all)	0.921	0.913
Precision (all)	0.953	0.926
Recall (all)	0.851	0.847
Knob mAP@50	0.961	0.952
Slider mAP@50	0.912	0.908
Pushbutton mAP@50	0.934	0.929
Touch mAP@50	0.882	0.873
Toggle mAP@50	0.915	0.902

**Table 12:** Segmentation performance metrics for each class, including precision, recall, and mean average precision at an IoU threshold of 0.5.

Class	Mask Precision (P)	Mask Recall (R)	Mask mAP@50
All	0.901	0.853	0.903
Knob	0.949	0.943	0.965
Slider	0.837	0.819	0.889
Pushbutton	0.937	0.876	0.932
Touch	0.887	0.779	0.877
Toggle	0.893	0.846	0.854

**Table 13:** Participant Characteristics.

Characteristic	Value
Number of participants	20
Age range	27–71 years
Main age group	Mostly 27–45 years
Participants aged 60 years or above	4
Self-reported color blindness	2
Recruitment strategy	Convenience sampling from the general population
Formal cognitive screening	Not performed
Formal visual acuity assessment	Not performed
Clinical diagnosis verification	Not performed

**Table 14:** Exploratory task-based comparison between the manual plus recipe-search condition and the AR application condition.

Measure	Manual + Recipe Search	AR Application	Observed Change
Mean completion time (min)	10.8	4.2	−6.6 min
Completion time range (min)	6.5–17.4	3.2–5.2	Shorter range
Task success rate	14/20 (70%)	19/20 (95%)	+25 percentage points
Mean observed errors	2.2	0.6	−1.6 errors
Mean perceived workload (0–100)	63.4	35.4	−28.0 points

**Table 15:** Mean Questionnaire Scores for Different Interaction Scenes.

Scene	Mean Score	Mean SD
Welcome and Introduction	4.53	0.50
QR Code Scanning	4.73	0.42
Appliance Detection and AR Overlays	4.70	0.45
Asking Questions and Visual Guidance	4.77	0.39
Overall Usability	4.75	0.43

## Data Availability

This study includes a custom dataset of appliance images and annotations collected from original captures, voluntary user submissions, and additional third-party image sources as described in the manuscript. Due to privacy, consent, and third-party usage restrictions, the full dataset is not publicly available. Publicly available datasets used as external resources are cited in the manuscript. Further information may be made available by the corresponding author upon reasonable request, subject to ethical and legal constraints.

## Abbreviations

The following abbreviations are used in this manuscript:

AR	Augmented Reality
AI	Artificial Intelligence
YOLO	You Only Look Once
YOLOv8	You Only Look Once, Version 8
YOLO-Seg	YOLO Segmentation Model
BoT-SORT	ByteTrack with Appearance Embedding Tracker
MCI	Mild Cognitive Impairment
SAM	Segment Anything Model
QR	Quick Response
ID	Identifier
PDF	Portable Document Format
API	Application Programming Interface
